# Zn-Doped Porous Graphitic Carbon Nitride: A High-Performance Catalyst for the Photodegradation of Pharmaceuticals and Personal Care Products

**DOI:** 10.1021/acsomega.5c04537

**Published:** 2025-09-08

**Authors:** Chidinma G. Olorunnisola, Damilare Olorunnisola, Christian Neumann, Wouter Koopman, Christina Günter, Harald Seitz, Harshadrai M. Rawel, Emmanuel I. Unuabonah, Andreas Taubert

**Affiliations:** † Institute of Chemistry, 26583 University of Potsdam, D-14476 Potsdam, Germany; ‡ African Centre of Excellence for Water and Environment Research (ACEWATER), 59199 Redeemer’s University, PMB 230 Ede, Osun State, Nigeria; § Department of Chemical Sciences, Redeemer’s University, PMB 230 Ede, Osun State, Nigeria; ∥ 28428 Fraunhofer-Institut für Angewandte Polymerforschung IAP, D-14476 Potsdam, Germany; ⊥ Institute of Physics and Astronomy, University of Potsdam, D-14476 Potsdam, Germany; # Institute of Geosciences, University of Potsdam, D-14476 Potsdam, Germany; ∇ Fraunhofer Institute for Cell Therapy and Immunology, Branch Bioanalytics and Bioprocesses (IZI-BB), D-14476 Potsdam, Germany; ○ Institute of Nutritional Science, University of Potsdam, Nuthetal, D-14558 Potsdam, Germany

## Abstract

In this study, photocatalytically active graphitic carbon nitride (GCN) photocatalysts with varying amounts of zinc salts were synthesized using a solvent-free, scalable, and eco-friendly method. The photocatalysts were characterized using X-ray powder diffraction, scanning electron microscopy, nitrogen sorption, thermogravimetric analysis, UV–vis diffuse reflectance, and time-resolved photoluminescence (TRPL) spectroscopy. The addition of zinc to the GCN yields materials (GCN:Zn) with an enhanced surface area up to ca. 150 m^2^/g. Moreover, Zn incorporation affects the electronic structure of GCN and improves the electron transfer rate in the materials. The GCN:Zn materials show significantly improved photocatalytic activity for the degradation of pharmaceutical and personal care products (PPCPs) such as sulfamethoxazole (SMX), tetracycline (TET), and triclosan (TC) compared to zinc-free GCN. Although the degradation efficiency exceeds 98%, mineralization of the PPCPs is moderate (45–53%) due to the formation of stable intermediates. The treated effluents are nontoxic to *Escherichia coli* and *Staphylococcus xylosus*, indicating that no harmful intermediates form during the photocatalytic degradation of the PPCPs. The GCN:Zn photocatalysts demonstrate excellent reusability and stability for the degradation of PPCP over four cycles with minimal loss in activity, showcasing their potential for sustainable water treatment applications.

## Graphical abstract

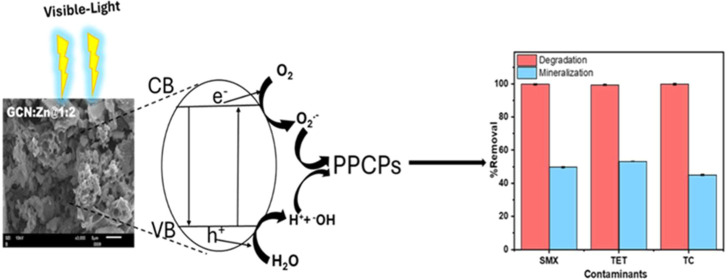

## 1 Introduction

The pervasive contamination of water bodies by pharmaceuticals and personal care products (PPCPs) has become a pressing environmental and public health issue. For instance, antibiotics such as sulfamethoxazole (SMX, Figure 1A) and tetracycline (TET, Figure 1B) are extensively used in human and veterinary medicine. They persist in aquatic environments and contribute to the emergence of antibiotic-resistant bacteria. This poses a significant threat to both ecosystems and public health.^1,2^ Similarly, triclosan (TC, Figure 1C), an antimicrobial agent commonly found in personal care products, is frequently detected in water systems. It accumulates and exerts toxic effects on aquatic life, disrupts endocrine systems, and may potentially bioaccumulate, leading to broader ecological implications.^3^

**Figure 1:**
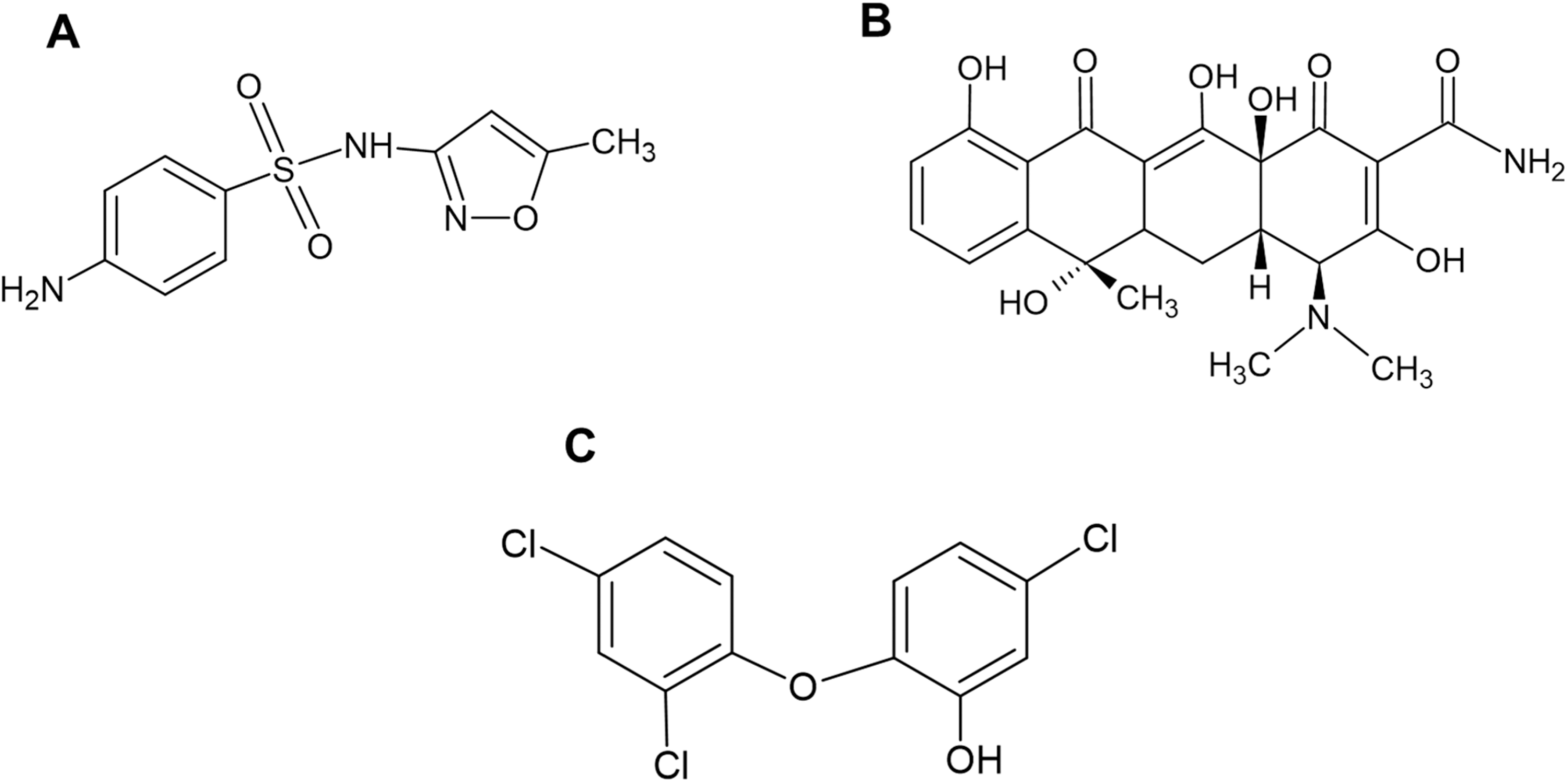
Chemical structure of (A) sulfamethoxazole (SMX), (B) tetracycline (TET), and (C) triclosan (TC).

Given the persistence of these contaminants, several studies have explored their degradation using advanced oxidation processes (AOPs), particularly photocatalysis, due to its efficiency in breaking down persistent organic pollutants.^4^ Among others, graphitic carbon nitride (GCN), which can be synthesized from nitrogen-rich precursors, such as Dicyandiamide, urea, and melamine through thermal condensation, has gained attention as a promising photocatalyst for the degradation of organic contaminants in water. This is due to its effective visible light absorption, low cost, and excellent thermal and chemical stability.^5,6^

However, despite its advantages, the photocatalytic activity of GCN is still limited owing to its low quantum yield and rapid recombination of photogenerated electron–hole pairs (Xing et al., 2021).^72^ To overcome these limitations, several methodologies have been employed to enhance the photocatalytic performance of GCN. Examples include loading known photocatalysts on the GCN surface, forming GCN nanohybrids, and doping the GCNs with transition metals.^7–9^ GCNs doped with transition metals such as silver, copper or zinc have demonstrated enhanced photocatalytic activity for the degradation of various pollutants, mostly by improving the charge separation efficiency and extending photocatalytic activity under visible light.^10,11^

Zinc chloride (ZnCl_2_) was selected as a dopant due to its ability to introduce ZnO and Zn^2+^ into the GCN structure while promoting porosity through gas evolution during calcination.^12^ The combination of GCN and zinc oxide (ZnO) has been recognized as an effective route to enhance charge separation in the electron transfer process and improve the photocatalytic performance. Recent studies have shown that ZnO/GCN composites exhibit significant improvements in their photocatalytic activity under visible light irradiation. For instance, Panthi et al. have reviewed strategies for the synthesis of GCN/ZnO-based Z-scheme and S-scheme heterojunction photocatalysts that enhanced the photocatalytic performance and stability of the photocatalyst for the degradation of organic pollutants.^13^ Paul et al. reported enhanced adsorption and photocatalytic activity of GCN-Zn composites over pure GCN for the degradation of methylene blue. These authors explained the improvement by the formation of GCN:ZnO heterojunctions, resulting in better separation of the photogenerated charge carriers.^14^ Additionally, Akintunde et al., reported the efficient photocatalytic degradation of organic pollutants using GCN/ZnO-Cu nanocomposites.^15^ The enhanced photocatalytic activity of the nanocomposite was attributed to the large surface area and the synergy of GCN and ZnO containing a few % Cu, resulting in an improved band gap.

However, despite these advancements, most studies rely on organic solvents or surfactants during the preparation of photocatalysts, which introduces additional environmental concerns. Only a few studies have explored solvent-free synthesis methods for metal-doped GCN composites.

In this study, a new and efficient solvent-free synthesis of porous GCN/Zn composites is described. SMX, TET, and TC were selected as target pollutants due to their widespread occurrence in water and their classification as persistent, recalcitrant micropollutants with known resistance to conventional treatment processes. These compounds represent structurally diverse PPCPs, enabling the evaluation of the versatility of the photocatalysts across different contaminant classes. The resulting materials are effective photocatalysts for the photodegradation of SMX, TET, and TC in water under visible light conditions. The synthesis approach, based on manual solid-state mixing without solvents, aligns with green chemistry principles by eliminating the need for harmful solvents and minimizing hazardous waste.

In this study, a new and sustainable solvent-free synthesis route for Zn-doped porous GCN composites offers a greener alternative to conventional solution-based methods. The selected target pollutants (SMX, TET, and TC) are commonly detected, structurally diverse PPCP that are known for their persistence in aquatic environments and resistance to conventional treatment technologies. The performance of the synthesized photocatalysts across these different classes of light was demonstrated. This work contributes to current research efforts by offering an efficient, scalable synthesis method aligned with green chemistry principles while advancing the application of metal-modified carbon nitride composites for water purification.

As a result, the approach simplifies catalyst preparation and offers a scalable and environmentally friendly route for photocatalyst fabrication. The incorporation of Zn into GCN enhances charge separation, thereby creating more active sites for the improved photocatalytic degradation of organic pollutants. Overall, the approach therefore combines a number of advantages, making it a promising strategy for sustainable water treatment technologies.

## 2 Materials and Methods

Melamine (C3N6H6, >99.9%), zinc chloride (ZnCl_2_), sodium hydroxide (NaOH), sulfamethoxazole (SMX, 98%), tetracycline (TET, >99%), and triclosan (TC, >99%) were purchased from Sigma-Aldrich and used without further purification. Deionized water was used throughout this study.

### 2.1 Preparation of the Photocatalyst

Pure graphitic carbon nitride (g-C_3_N_4_, GCN) was synthesized by heating melamine in a program-controlled furnace. In a typical synthesis run, 10 g of melamine were placed in an alumina crucible with a lid. The covered crucible was placed in the oven and heated to 550 °C at a heating rate of 5 °C/min. Holding time at 550 °C was 1 h, and then the oven was allowed to cool to room temperature.

The preparation of the GCN:Zn photocatalyst was carried out via manual solid-state mixing using 3 g of melamine, 3 g of ZnCl_2_, and 1.5 g of NaOH. The precursors were placed in a mortar and manually mixed with a pestle until a uniform mixture was obtained. Thereafter, the mixture was placed in an oven at 65 °C overnight. Afterward, the powder was calcined in a muffle oven using the same conditions as described for the synthesis of the neat GCN above. In this study, different photocatalysts were prepared, where the weight fractions of GCN and ZnCl_2_ were varied during the photocatalyst synthesis, and the weight of the NaOH was kept constant, as seen in Table 1. For simplicity, the obtained GCN:Zn composites with different contents of both GCN and Zn were labeled as GCN-*x*/Zn-*x*, where *x* represents the GCN or Zn weight fraction in the composites.

**Table 1:** Description of the Different Components Used in the Preparation of the Photocatalysts Used in This Study

materials (labels)	*W*_t_ of melamine (g)	*W*_t_ of ZnCl_2_ (g)	*W*_t_ of NaOH (g)	temperature (°C)
GCN	10			550
GCN:Zn@2:1	6	3	1.5	550
GCN:Zn@1:1	3	3	1.5	550
GCN:Zn@1:2	3	6	1.5	550

### 2.2 Instrumental and Characterization

Powder X-ray diffraction (PXRD) was done on a PANalytical Empyrean Powder X-ray diffractometer (Malvern, U.K.) in a Bragg–Brentano geometry equipped with a PIXcel1D detector using Cu Kα radiation (λ = 1.5419 Å) operating at 40 kV and 40 mA; θ/θ scans were run from 4 to 70° 2θ with a step size of 0.0131° and a sample rotation time of 1 s.

Scanning electron microscopy (SEM) was performed on a JEOL JSM-6510 SEM (Freising, Germany) equipped with an Oxford (INCAx-act SN detector) energy dispersive X-ray (EDX) detector.

Elemental analysis (EA) was done on an Elementar Vario EL III (Langenselbold, Germany) instrument in duplicate.

Attenuated total reflectance Fourier transform infrared (ATR-FTIR) spectroscopy was done on a Nicolet iS5 (Thermo Scientific, Waltham, MA, iD7 ATR unit with a diamond crystal, resolution of 4 cm^–1^, 32 scans, from 400 to 4000 cm^–1^).

The point of zero charge, pH_pzc_, was determined via the salt addition method, providing the net surface charge of the photocatalyst.^1^

Nitrogen sorption measurements were done on a Microtrac Belsorp MAX (formerly from BEL instruments) and Microtrac Belsorp MINI X. The samples were prepared and activated in a Microtrac BELPREP VAC III vacuum degasser (24 h under vacuum at 150 °C). The specific surface area was calculated by using the Brunauer–Emmett–Teller (BET) model. The average pore sizes were estimated from the adsorption branch of the isotherm by using the Barrett–Joyner–Halenda method (BJH). The pore volume was determined at *P*/*P*_0_ > 0.99.

The particle size distribution was measured with an LS 13 320 XR particle size analyzer (Beckman Coulter) with a measurement range between 0.04 and 2000 μm. The polarization intensity differential scattering (PIDS) technology was used to estimate the particle sizes in the subμm range. The samples were measured in deionized water in a universal liquid module (ULM). The samples were incrementally added to the module until optimal obscuration, indicated by the device, was reached. All samples were measured three times and averaged

UV–vis diffuse reflectance spectra (UV–vis DRS) were collected on a PerkinElmer Lambda 950 UV–vis spectrophotometer with the solid material attachment Praying Mantis (Harrick Scientific Products Inc.). Magnesium sulfate (MgSO_4_) AnalaR NORMAPUR by VWR (Leuven, Belgium) was used as a background material. The measuring range was λ = 200–850 nm with a resolution of 2 nm. The measured reflection data *R* were converted to the K/S ratio according to the Kubelka–Munk function, eq 1.^16^

1 $$\frac{K}{S}=\frac{{\left(1-R\right)}^{2}}{2R}$$

In eq 1, *K* is the absorption coefficient, *S* is the scattering coefficient, and *R* is the reflectance. From these converted data, the optical band gaps were graphically analyzed using the Tauc analysis.

2 $${\left(\alpha \mathrm{hv}\right)}^{2}=A\left(\mathrm{hv}-E_{g}\right)$$

where α, *h*ν, *A*, and *E*_g_ are the absorption coefficient, photon energy, constant, and band gap energy, respectively. By linear extrapolation of (α*h*ν)^2^ vs *h*ν, the band gap energy can be obtained.

Ultrafast time-resolved photoluminescence (TRPL) measurements were performed by employing a streak camera setup. In short, femtosecond laser pulses (150 fs) with a central wavelength of 800 nm and a repetition rate of 5 kHz were produced by chirped pulse amplification (Spectra Physics Spitfire). Subsequently, the third harmonic of the fundamental frequency at 266 nm was generated by two-step nonlinear frequency conversion (Eksma optics Femtokit) and used to excite the samples. The excitation power was fixed to 2.2 mW. Finally, the time-resolved PL was measured by using a streak camera (Hamamatsu C5680 + M5677) with a temporal resolution of 250 ps. The background signal was corrected to avoid the influence of scattering and environmental light. To increase the temporal resolution, the instrument response function was determined and deconvolved during the fitting procedure.

Thermogravimetric analysis (TGA) was done on a Mettler Toledo instrument (TGA/DSC 3+) from 25 to 900 °C under nitrogen (50 mL/min gas flow) with a heating rate of 10 K/min.

Liquid chromatography-mass spectrometry (LC–MS)/MS analysis was performed using Agilent G6470A Series Triple Quad LC/MS (Agilent Technologies Sales & Services GmbH & Co.KG, Waldbronn, Germany) and HPLC Agilent Infinity 1260 System (binary pump, multicolumn thermostat, vial sampler VL, UV–vis Dual Wavelength Detector) (set at 470, 538 nm).

### 2.3 Photocatalytic Degradation of Contaminants

The photocatalytic activity of the GCN:Zn composites was evaluated using the selected PPCPs (SMX, TET and TC) in a custom-built photoreactor equipped with Philips Masters PL-L 4p 36W/840 fluorescent lamps as the light source. The detailed configuration of this photoreactor has been previously described.^17^ For the photodegradation experiment, an initial kinetic study to determine the efficiencies of the prepared photocatalysts was carried out by exposing 100 mL of 10 mg/L solutions of the contaminants (SMX, TET, and TC) to 50 mg of the GCN:Zn photocatalysts. The mixture was agitated for 120 min, and aliquots of 1 mL were withdrawn during the experiment at different time intervals using a predetermined time program. The aliquots withdrawn were immediately filtered with 0.2 μm polytetrafluoroethylene (PTFE) syringe filters, and residual PPCP concentrations were analyzed and quantified using the Agilent G6470A Series Triple Quad LC/MS described above. Untreated water samples containing identical concentrations of PPCP were also passed through the PTFE filters to identify errors induced by the adsorption of PPCP on the filters. A control photolysis study was conducted under identical experimental conditions as described above but in the absence of the GCN:Zn photocatalyst.

The degree of photocatalytic degradation was calculated from eq 3:

3 $$\%\;\mathrm{degradation}=\frac{C_{0}-C_{t}}{C_{0}}\times 100$$

where *C*_0_ and *C*_t_ represent the initial and final concentrations for the LC analysis after treatment with the composite, respectively.

The degree of mineralization in water with the most effective photocatalyst (GCN:Zn@1:2) was determined via the measurement of the total organic carbon (TOC) of the treated and untreated water using a Vario TOC analyzer (TOC/TNb Analyzer, vario TOC cube, Elementar Analysensysteme GmbH, Hanau, Germany). The % mineralization was calculated using eq 4.

4 $$\%\;\mathrm{mineralization}=\frac{(\mathrm{TO}C_{o}-\mathrm{TO}C_{e})}{{\mathrm{TOC}}_{o}}\times 100$$

where TOC_o_ and TOC_e_ refer to the initial and the final total organic carbon respectively.

### 2.4 Effect of Process Variables

The influence of operating variables on PPCP degradation was studied by using the catalyst with the best efficiency (GCN:Zn@1:2). First, 100 mL of 10 mg/L PPCP solutions were irradiated for 60 min using a photocatalyst dose GCN:Zn@1:2 of 15, 25, 50, 75, and 100 mg. The solution pH was varied from 3.0, 5.0, 7.0, 9.0, and 11.0, and adjusted with 0.1 M HCl and 0.1 M NaOH. The effect of initial PPCP concentration (2, 5, 15, and 20 mg/L), ionic strength of the PPCPs solution (0.01M, 0.025, and 0.05 M NaCl and MgCl_2_) and anions (1.0 mM of Cl^–^, SO_4_^2–^, CO_3_^2–^, PO_4_^3–^) on the photocatalytic degradation of 50 mL of the target PPCPs using 25 mg of GCN:Zn@1:2 were also investigated.

The stability and reusability of the best performing photocatalyst GCN:Zn@1:2 was studied by treating 100 mg of GCN:Zn@1:2 with 100 mL of a 10 mg/L PPCP solution for 60 min under visible light irradiation. After each experiment, the photocatalyst was recovered, rinsed with water, and dried in an oven at 60 °C before reuse.

### 2.5 Identification of Reactive Species and Degradation Products

To investigate the main reactive species generated upon irradiation different scavengers were used. One mM sodium oxalate (Na_2_C_2_O_4_, a hole (h^+^) scavenger), 0.2 mL isopropanol (IPA, an ^•^OH scavenger), or 0.5 mM benzoquinone (BQ, a superoxide scavenger). All measurements were taken in duplicate.

Identification of the degradation products was done via high-performance liquid chromatography–mass spectrometry (HPLC-MS, Agilent Infinity HPLC 1260 System using Agilent G6470A Series Triple Quad LC/MS, Agilent Technologies Sales & Services GmbH & Co.KG, Waldbronn, Germany) using solutions that were irradiated for 120 min in the presence of the photocatalyst. A Poroshell 120 EC-C18 Column (3.0 × 50 mm, 2.7 μm, Agilent Technologies Sales & Services GmbH & Co.KG, Waldbronn, Germany) was employed for the separation. The binary mobile phase for SMX and TET consisted of 0.1% formic acid (solution A) and methanol (solution B) at a flow rate of 0.6 mL/min. The elution gradient for the mobile phase is as follows: Gradient: 0 min—100% solution A, 2 min—100% solution A, 2–12 min; 100–50% solution A, 12–13 min; 50–5% solution A, 13–15 min, 5% solution A; 15–16 min, 5–100% solution A; 16–18 min, 100% solution A; The difference to 100% is solution B. Mass spectrometry analysis was carried out using a triple-stage quadrupole mass spectrometry. Samples were injected in the selected reaction monitoring (SRM) mode, using multiple reaction monitoring (MRM). The column temperature was 25 °C. Analyses were performed in electron spray ionization (ESI) positive/negative ion mode using the following settings: nebulizer pressure of 35.0 psi, and a desolvation nitrogen gas at a flow rate of 11.0 L/min. MS scans were performed in the range = 100–1000 *m*/*z* at fragmentor voltage = 80 V with a scan time = 40 ms.

### 2.6 Toxicity of Treated Water

Agar-disc diffusion assays were applied to determine whether the degradation products still retain antibiotic activity and to follow the effects of TET degradation over treatment (i.e., irradiation) time. In order to compare the relative toxicities of TET and aliquots from treated water at different time intervals, the change in toxicity against TET-sensitive bacteria (Gram-positive *Staphylococcus xylosus* and Gram-negative *Escherichia coli* SCS1) was monitored. One colony of each bacterial strain was cultured in 20 mL of 2YT medium and incubated overnight at 37 °C while shaking at 100 rpm/min (called “overnight culture”). Subsequently, 20 mL of 45 °C warm 2YT agar was mixed with 10 μL of the overnight culture to prepare the plate. From each of the test samples (the parent compound and the aliquots taken at specific time intervals), 500 μL were taken and lyophilized down to 50 μL. The necessary number of paper disks (4.5 mm) was deposited on the bacteria agar plate, and 25 μL of 10x concentrated process samples were pipetted on each disc. TET was used as a positive control, and distilled water with 5% ethanol was used as a negative control. The plate was incubated overnight at 37 °C and evaluated. The inhibition zone diameter was used as a criterion to determine the antimicrobial toxicity.

## 3 Results and Discussion

### 3.1 Material Characterization

Figure 2A shows the representative ATR-FTIR spectra of all materials. In the spectra of neat GCN, the sharp band at 808 cm^–1^ corresponds to the out-of-plane bending vibration characteristic of triazine rings.^18^ This signal has been previously assigned to the presence of the heptazine structure in GCN,^19^ and it is present in all photocatalyst composites. The bands between 1200 and 1650 cm^–1^ are associated with the stretching modes of GCN heterocycles, which are characteristics of the two-dimensional (2D) carbon nitride framework.^20^ The bands at 1241, 1313, 1406, and 1637 cm^–1^ in the spectra of GCN are attributed to the aromatic C–N stretching,^21^ while the broad peak at around 3100 cm^–1^ is assigned to the stretching vibration of –NH and hydroxyl groups present in the GCN structure.^22^

**Figure 2:**
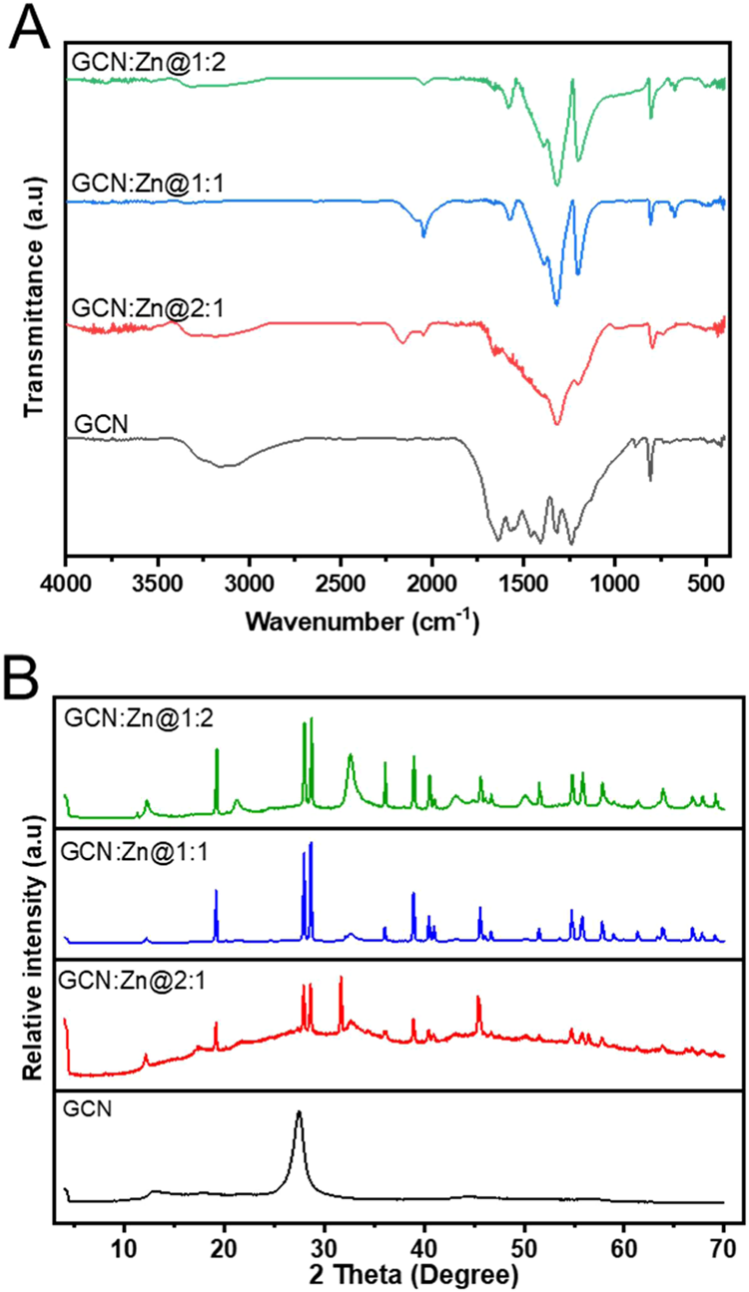
(A) FTIR spectra and (B) XRD data of GCN and all photocatalysts used in this study.

In the case of the other photocatalysts (GCN:ZN@2:1, GCN:ZN@1:1, and GCN:ZN@1:2), the introduction of Zn ions modifies the bands in the 1200–1650 cm^–1^ region, reflecting changes in the chemical bonding and interactions between Zn and the nitrogen or carbon atoms in GCN. The broad band at around 3100 cm^–1^ in all photocatalyst composites is assigned to the stretching vibration of –NH and hydroxyl groups. This peak is weakened as the amount of Zn increases. This suggests modifications of the hydrogen bonding network in the composite upon Zn incorporation. The appearance of a band at 670 cm^–1^ in GCN:Zn@1:1 and GCN:Zn@1:2 corresponds to Zn–O or Zn–N bonds, which indicate the interaction between Zn and the nitrogen or carbon atoms in GCN.^23^

Figure 2B shows the X-ray diffraction (XRD) patterns of pristine GCN and Zn-doped GCN photocatalysts. The pure GCN shows two characteristic diffraction signals. The intense (002) reflection at 27.5° results from the interplanar stacking peak of the aromatic layers in GCN.^4,24^ The less intense (100) reflection at 13.1° results from the in-plane packing of tris-triazine units. This latter reflection is present in all photocatalysts, indicating that the graphitic-like packing motif remains intact^25^ upon addition of Zn.

Compared to GCN, the appearance of many peaks in the diffractograms of the Zn-doped GCN samples suggests the distortion in graphitic stacking, and thereby the crystal structure is affected by the introduction of Zn. A similar observation has been reported in the literature.^4,26^

All prepared composites with Zn show similar diffractograms with sharp reflections seen at 27.9, 28.6, 38.9, 40.5, 45.6, 54.9, and 56.0°. These signals align with the reference diffraction pattern of zinc cyanamide (ICSD 98-028-0523), which is provided in the Supporting Information (Figure S1). This could possibly be from the reaction between zinc species and N-rich organics (melamine) used in the preparation of the composites. While it is unlikely that zinc cyanamide is formed in the reaction, the resulting material seems to have a rather high structure or similarity with zinc cyanamide. This could be rationalized by the fact that the cyanamide ion can be viewed as “cut-out” of the GCN structure. Overall, the XRD data do not allow for a clear assignment of the state of Zn in the materials.

The presence of these additional peaks may also indicate the formation of intermediate zinc–nitrogen species. However, no distinct peaks corresponding to melamine were observed, likely due to the high thermal treatment temperature (550 °C), which exceeds the reported decomposition and polymerization point of melamine as previously reported in literature.^27,28^

Figure 3 shows scanning electron microscopy (SEM) images of the photocatalysts. The morphology of GCN (Figure 3A) displays an irregular, stacked sheet-like structure typical of GCN with noticeable agglomeration and two-dimensional lamellar structures.^25^ The introduction of Zn (Figure 3B–D) significantly alters the surface morphology of GCN, resulting in rougher, more irregular surfaces and the appearance of small, aggregated clusters, particularly in the higher Zn-loading samples. These clusters may indicate localized Zn-rich domains or structural rearrangements during the synthesis.

**Figure 3:**
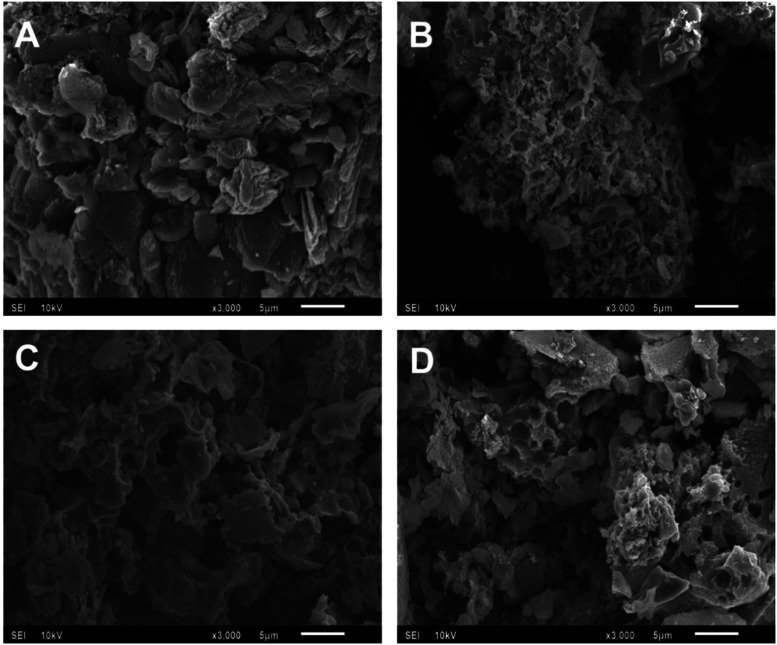
Scanning electron image (A) GCN, (B) GCN:Zn@2:1, (C) GCN:Zn@1:1, and (D) GCN:Zn@1:2.

The Zn-containing composites also exhibit visible void-like features, which is probably due to the influence of gases released from the precursor decomposition and condensation during the thermal polymerization reaction.^29^ Notably, Figure 3D shows a more open structure with particles that are slightly smaller than the particles in the other materials, but this is very hard to judge from the SEM images alone; particle size distribution analysis (Figure 5, below), however, seems to support that observation. The presence of these rougher surfaces and separated particles in Figure 3D suggests an increased textural openness, which may enhance the surface area and provide more active sites. This may be beneficial for photocatalysis.

Figure 4A shows the thermogravimetric analysis (TGA) data of GCN and the GCN:Zn composites. GCN shows an initial slight weight loss of 2% between 28 and 198 °C, which is assigned to the loss of some residual water and surface-adsorbed species. This is followed by a sharp weight loss of approximately 98% between 550 and 760 °C, which is due to the (likely oxidative) decomposition of GCN in the air atmosphere used for these experiments.^30^ At ca. 760 °C, full combustion is achieved.

**Figure 4:**
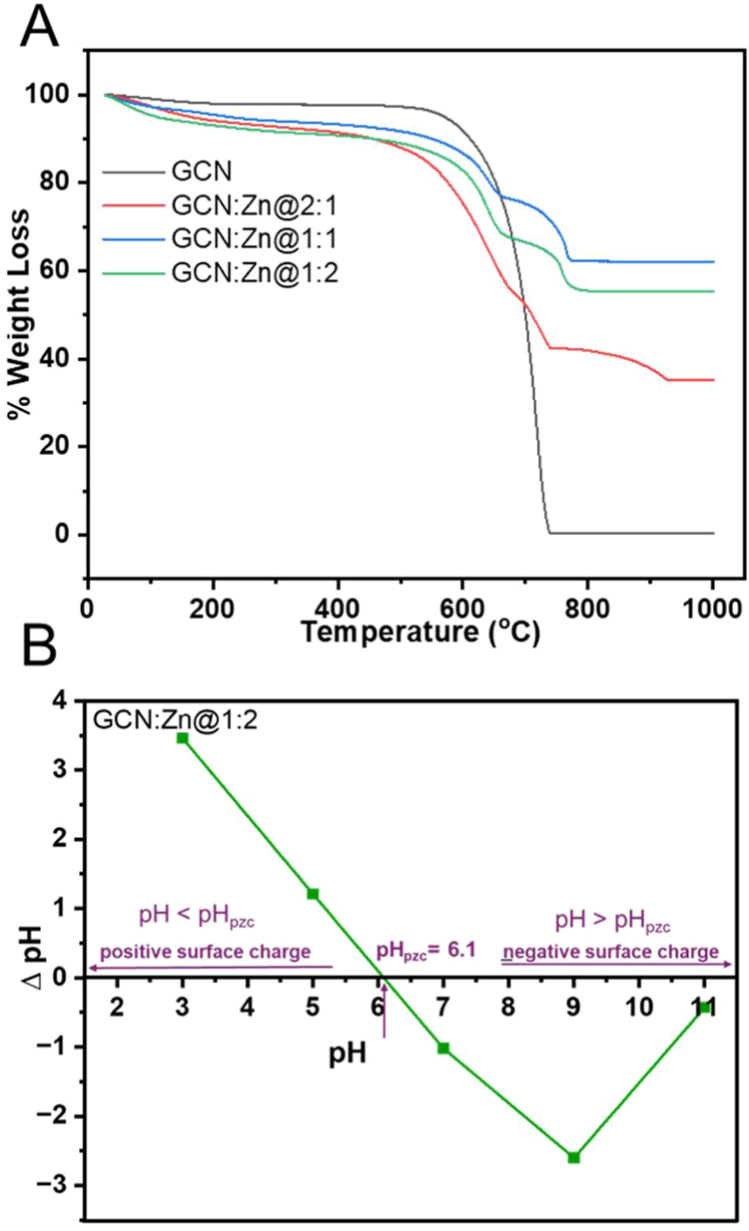
(A) TGA data of all photocatalysts and (B) pH_pzc_ plot obtained for GCN:Zn@1:2.

In contrast to GCN, which essentially shows a one-step decomposition, GCN:Zn@2:1 shows a four-step decomposition, with an initial weight loss of 5% between 27 and 167 °C, which is related to the loss of some residual water. The second weight loss of ca. 33% is between 480 and 675 °C, and it is associated with the decomposition of the GCN phase in the composite. The third weight loss of ca.13% is between 676 and 739 °C, and this could be attributed to the presence of Zn in the composite. The fourth weight loss of ca. 7% is between 740 and 927 °C, could be attributed to the final decomposition of thermally stable phases of GCN

However, GCN:Zn@1:1 and GCN:Zn@1:2 show a three-step decomposition, with an initial weight loss ca. 5% between 28 and 170 °C, which is due to loss of water. Between 500 and 680 °C, weight losses of ca. 19 and 26% are observed for GCN:Zn@1:1 and GCN:Zn@1:2, respectively. This weight loss is associated with the decomposition of the GCN phase in the composites. The last weight loss of about 15% in these composites, which is observed between 680 and 800 °C, could be attributed to the presence of Zn, which could potentially facilitate further decomposition at this temperature range.

Figure 4B shows the point of zero charge, pH_pzc_ of GCN:Zn@1:2, a critical property that defines the pH at which the surface of the material carries no net electrical charge. This value plays a key role in the interaction of the photocatalyst with charged species in solution and influences its overall catalytic activity, because Coulomb attraction or repulsion between the catalyst and the pollutant may strongly influence the outcome of a catalytic reaction. The pH_pzc_ of GCN:Zn@1:2 is 6.1, meaning that at pH values below 6.1, the surface of GCN:Zn@1:2 is positively charged, which usually enhances the adsorption of negatively charged species from solution. Conversely, at pH values above 6.1, the surface is negatively charged, favoring the adsorption of positively charged species.

The specific surface areas and porosities of the photocatalysts were evaluated via nitrogen sorption (Table 2 and Figure S2). The isotherms of the materials (Figure S2) display type IV behavior with a clear hysteresis loop, which is typical of mesoporous materials.^26,31^ Table 2 shows that the surface area varies significantly among the different materials. The BET surface area for GCN:Zn@1:2 is the highest, measured at 150.2 m^2^/g, followed by that for GCN:Zn@1:1 with 63.7 m^2^/g. These values are substantially higher than those of pure GCN and GCN:Zn@2:1, which exhibit much lower surface areas of 14.0 and 14.7 m^2^/g, respectively. A similar observation has been reported by other authors.^4,14^

**Table 2:** Nitrogen Sorption Data and Elemental Analysis of the Photocatalysts

				elements (% composition)			
sample	*S*_BET_ (m^2^/g)	pore volume (cm^3^/g)	pore diameter (nm)	C	H	N	C/N
GCN	14.0	0.12	36.65	35.86	1.58	62.81	0.57
GCN:Zn@2:1	14.7	0.03	8.91	20.33	0.30	35.08	0.58
GCN: Zn@1:1	63.7	0.09	5.74	15.11	0.09	28.98	0.52
GCN:Zn@1:2	150.2	0.22	5.95	14.99	0.46	26.83	0.56

The significant increase in surface area upon zinc incorporation suggests enhanced porosity, which can potentially create more active surface sites. This confirms the mesoporous nature of the synthesized materials, especially in the GCN composites,^32^ where pore sizes fall within the mesopore range (2–50 nm). The hysteresis loop observed in GCN:Zn@1:1 and GCN:Zn@1:2 is more pronounced than in GCN or GCN@2:1, suggesting greater pore volume and better developed mesoporosity.

Furthermore, it has been reported that materials with higher crystallinity tend to exhibit smaller pore diameters due to their more ordered atomic structure, which restricts the formation of larger, irregular pores.^33^ This aligns with the observed pore diameters of 5.67 nm for GCN:Zn@1:1, and 5.8 nm for GCN:Zn@1:2. The smaller pore diameters in GCN:Zn@1:1, and GCN:Zn@1:2 indicate enhanced crystallinity. When combined with the increased surface area, this also suggests that Zn incorporation significantly improves the microstructure and porosity of the materials.

Table 2 shows the elemental compositions of the photocatalysts. The highest amount of carbon and nitrogen is found in the GCN material, which is characteristic of a material primarily made up of C–N bonds. The theoretical C:N ratio for an ideal GCN (3:4) is 0.75. However, from the elemental analysis result shown in Table 2, the synthesized GCN revealed a lower C:N ratio of 0.57, suggesting a nitrogen-rich structure. This deviation likely arises from the incomplete polymerization of the precursor (melamine) during synthesis. Interestingly, the C:N ratio remains unchanged upon Zn doping, indicating that the introduction of Zn species does not significantly alter the elemental composition of GCN. However, the addition of Zn species partially replaces C and N atoms, lowering their overall percentage in the material.^14^

The particle size distribution (Figure 5) of the photocatalysts shows that GCN and GCN:Zn@2:1 have larger particle sizes compared to those of GCN:Zn@1:1 and GCN:Zn@1:2, which exhibit smaller, more uniform particle sizes.

**Figure 5:**
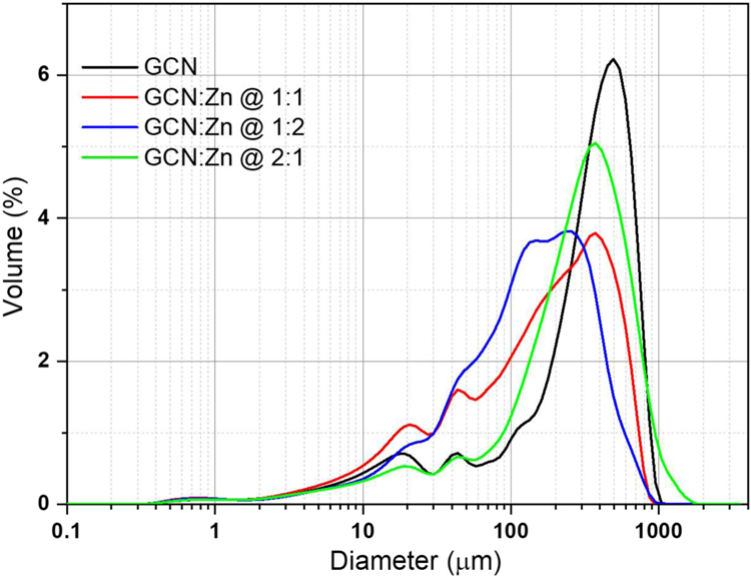
Particle size distribution of all photocatalysts.

The optical properties of the photocatalysts were investigated by using solid-state UV–vis diffuse reflectance spectroscopy (UV–vis DRS). The UV–vis DRS spectrum of GCN shows an absorption band edge around 450 nm (Figure 6A), which is typical of graphitic carbon nitride, and confirms the ability of GCN to absorb visible light.^34,35^ Upon zinc doping, the absorption band edge of GCN:Zn@1:1 and GCN:Zn@1:2 (ca. 395 nm) are the same regardless of the amount of Zn added, and they shift toward shorter wavelengths, indicating a blue shift in absorption.

**Figure 6:**
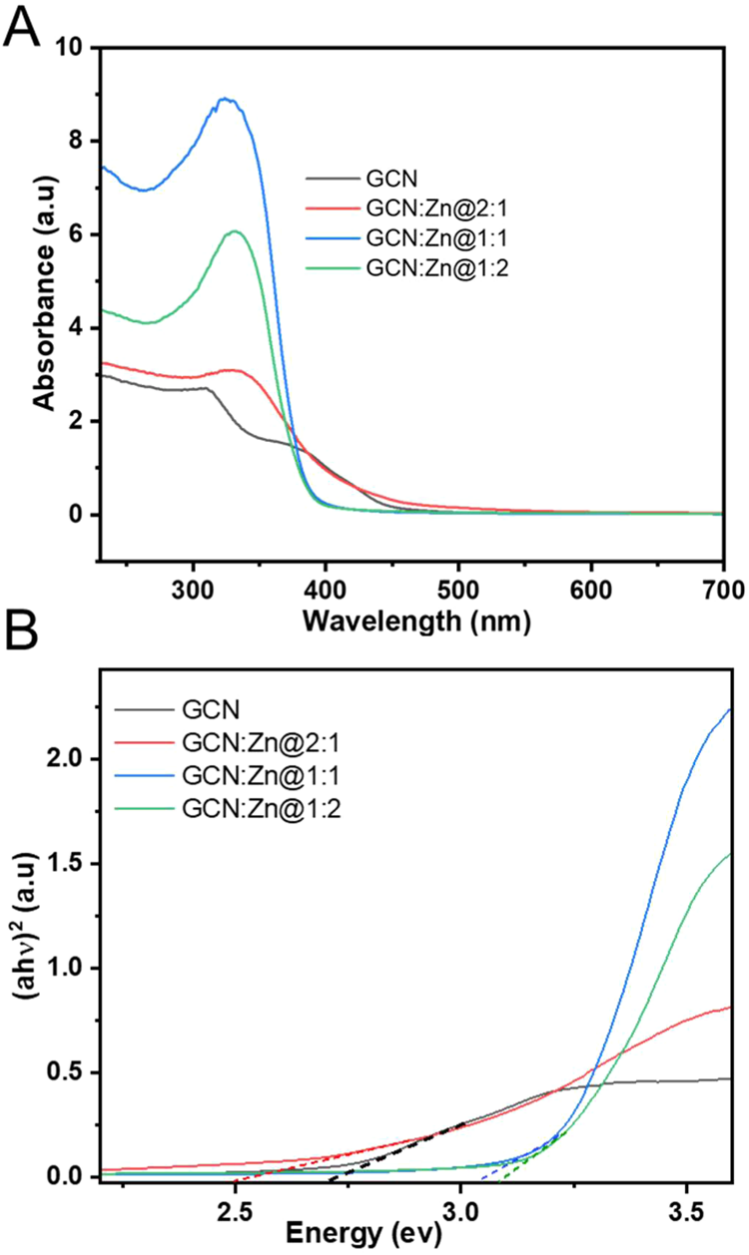
(A) UV–vis DRS spectra and (B) Tauc plot analysis of all of the prepared photocatalysts.

To understand this blue shift, it is important to examine the molecular structure. GCN acts as an n-type semiconductor, where the -NH/NH_2_ groups in its structure can act as electron donors that contribute free electrons to the conduction band.^36^ However, the addition of Zn to GCN appears to alter the electronic structure of GCN. This causes an intramolecular charge transfer between the electron-rich -NH/NH_2_ groups and the Zn­(II) ion.^37^ Additionally, the incorporation of Zn leads to the formation of a heterojunction (likely a GCN/Zn@GCN heterojunction because the Zn may not be distributed homogeneously in the material), which supports an effective charge separation and reduces the recombination of the electron–hole pairs in the composite.^4^

The band gap energies were calculated from the Tauc plots, Figure 6B.^38^ GCN and GCN:Zn@2:1 exhibit the lowest band gaps of 2.7 and 2.5 eV, respectively. GCN:Zn@1:1 and GCN:Zn@1:2 exhibit band gaps of 3.04 and 3.08 eV respectively. This is consistent with the blue shift observed in the UV–vis DRS spectra. As a result, GCN and GCN:Zn@2:1 effectively absorb visible light, while the other two photocatalysts absorb mostly in the UV region. This indicates that the incorporation of Zn, which apparently acts as an electron-withdrawing center, modifies the electronic structure of GCN and leads to an increased band gap.

Figure 7A shows the time-integrated PL spectra of all photocatalysts. The dip in the spectrum is caused by a notch filter blocking the scattered residual second harmonic radiation (400 nm). The PL maximum for GCN is observed at 460 nm, which is in accordance with previously reported literature values.^39,40^ When adding Zn, the weight of the PL spectrum moves to higher energies (lower wavelength) for higher Zn content. This confirms the larger band gap for increasing Zn content obtained from the UV–vis DRS spectra. The transients of the spectrally integrated data, presented in Figure 7B, exhibit a biexponential decay for all photocatalysts, characterized by a short lifetime (τ_1_) and a long lifetime (τ_2_). The short τ_1_ component in GCN is likely associated with the π-π* transition of the sp^2^-bonded core carbon in the heptazine ring, while the long τ_2_ component is attributed to the presence of intragap states.^40^ Upon Zn doping, the lifetimes are reduced to roughly half the value of GCN, indicating an efficient additional nonradiative channel.^41^ We speculate that charge transfer to Zn atoms in the composite photocatalysts (GCN:Zn@2:1, GCN:Zn@1:1, and GCN:Zn@1:2) is responsible for additional quenching. This very fast electron transfer could explain the improved photocatalytic efficiency of the doped materials versus pure GCN.

**Figure 7:**
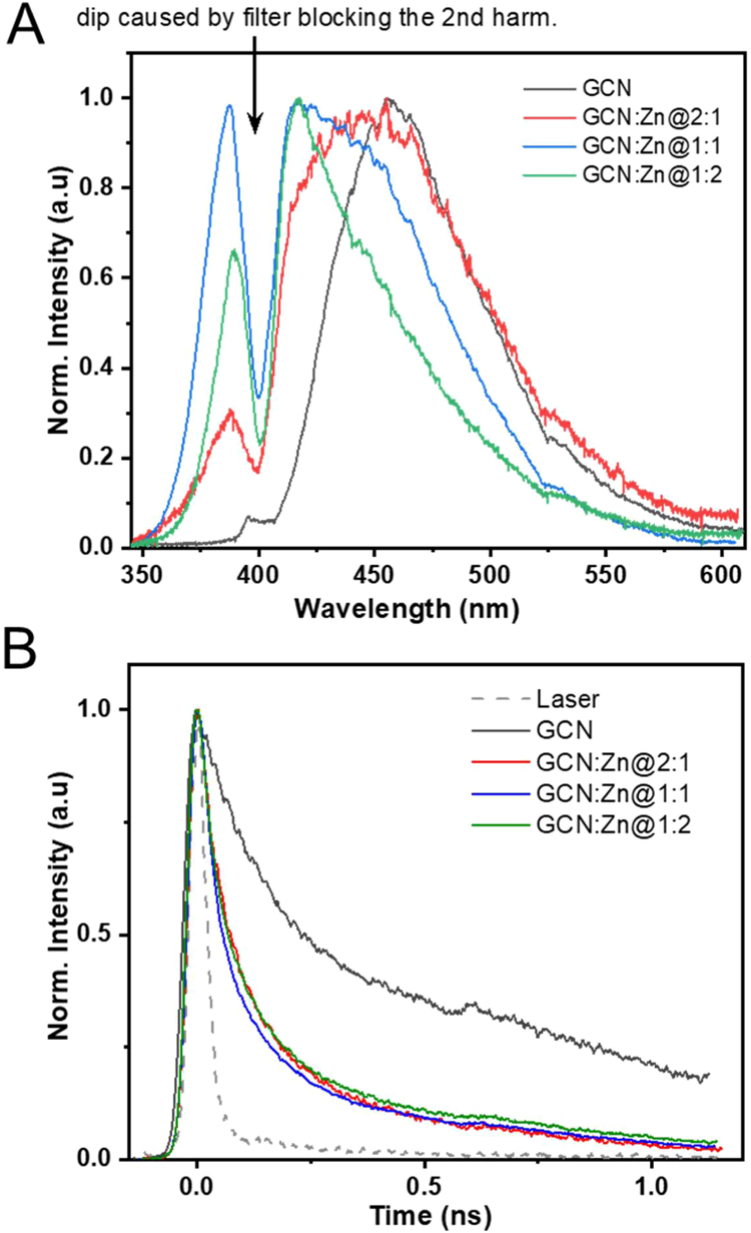
(A) Time-integrated photoluminescence spectroscopy after UV excitation at 266 nm with 2 mW and (B) The spectrally integrated TRPL transients of all prepared photocatalysts.

### 3.2 Photocatalytic Activity of the Composites

The photocatalytic composites were first screened for their photocatalytic efficiency for the degradation of PPCPs (SMX, TET, and TC) in water under visible light conditions provided by the fluorescent lamps used in the setup. First, the effect of direct photolysis was determined in the absence of the photocatalysts. The degradation of SMX, TET, and TC by photolysis is negligible within the time frame of the experiment (Figure 8A–C).

**Figure 8:**
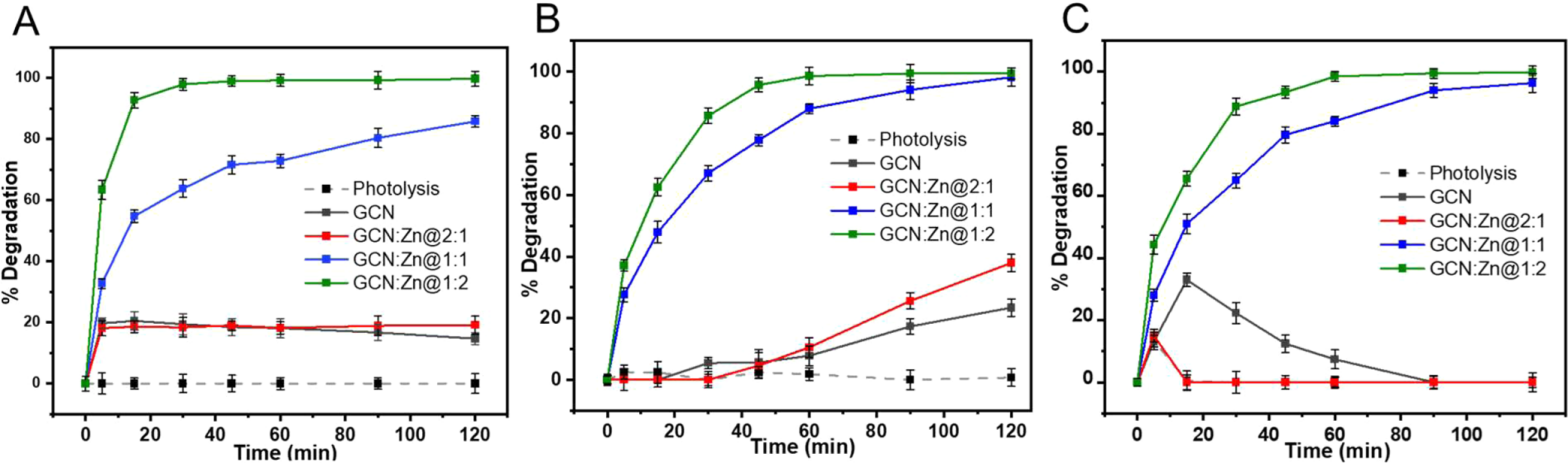
Degradation of (A) SMX, (B) TET, and (C) TC using GCN, GCN:Zn@2:1, GCN:Zn@1:1, and GCN:Zn@1:2, along with the control photolysis experiment (i.e., no catalyst is present).

In contrast, solutions treated with GCN (Figure 8A–C) show a different behavior: after 120 min SMX, TET, and TC are degraded to 0, 23, and 7%, respectively. This behavior is also observed with GCN:Zn@2:1 photocatalyst, where after 120 min SMX, TET, and TC are degraded to 19, 37, and 0% respectively. On the other hand, after 120 min GCN:Zn@1:1 degraded SMX, TET, and TC to 85, 98, and 96%, respectively. In contrast, GCN:Zn@1:2, shows 99% degradation of all contaminants (SMX TET, and TC) after 120 min. The high efficiency exhibited by GCN:Zn@1:2 is attributed to its higher surface area, as shown in Table 2, which likely provides more active sites for photocatalytic reactions.

For the remainder of this study, we focus on one material, GCN:Zn@1:2, because this photocatalyst has the highest degradation efficiency for the target contaminants of this study.

### 3.3 Effect of Operating Variables on PPCP Degradation

Photocatalytic activity is usually influenced by environmental parameters such as initial pollutant concentration of target contaminants, catalyst dose, pH, ionic strength, and anion type. The effects of various operating variables on the photocatalytic degradation of SMX, TET and TC using GCN:Zn@1:2 are presented below.

The effect of the catalyst dose on PPCP degradation was investigated by varying the amount of GCN:Zn@1:2 in solutions with a fixed PPCP concentration of 10 mg/L. Figure 9 shows a significant increase in the PPCP degradation efficiency as the catalyst dose increases from 15 to 100 mg. This improvement is attributed to the availability of more active sites on the catalyst surface, which enhances the generation of reactive oxygen species (ROS), which in turn leads to a more effective oxidative degradation of the pollutants, similar to previous data.^42^ In addition, the speed of degradation also improves with increasing catalyst dose from 15 to 100 mg. For instance, at 30 min ca. 98% degradation is achieved for SMX (Figure 9A) using 50 mg, 75 mg, and 100 mg. In the case of TET (Figure 9B), 85% degradation is achieved using 50 mg at 30 min, while complete degradation (100%) is achieved when both 75 and 100 mg of catalysts are used. For TC (Figure 9C) at 30 min, 88% degradation is achieved using 50 mg, and ca. 97% degradation is achieved for both 75 and 100 mg of catalysts. Whereas, at 60 min, 100% removal of all contaminants (SMX, TET, and TC) is achieved using 50, 75, or 100 mg of catalyst. However, only in the case of TET, 25 mg of the catalyst are sufficient to completely degrade TET within 60 min.

**Figure 9:**
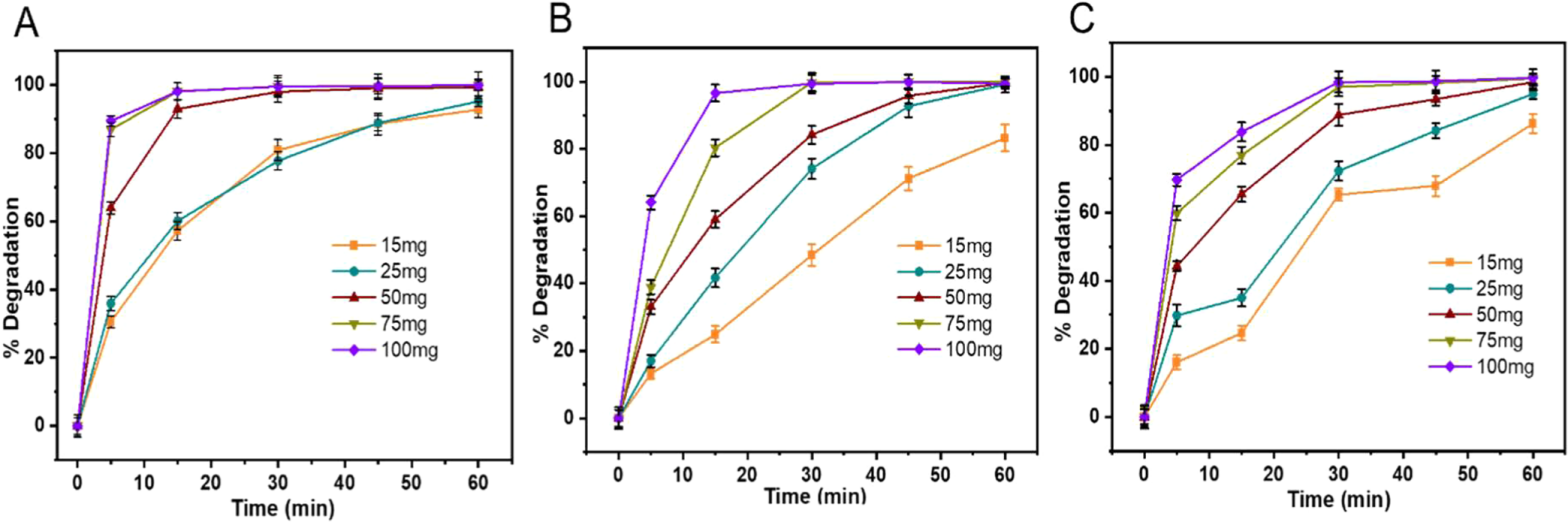
Effect of photocatalyst dose on (a) SMX, (b) TET, and (c) TC.

Notably, at lower doses (15 and 25 mg), degradation occurs at a slower rate, requiring a longer reaction time to reach high degradation efficiencies. The faster degradation observed at higher catalyst doses is likely due to the increased density of active sites, which accelerates the formation of ROS and enhances pollutant breakdown within a shorter time frame.

Therefore, to avoid the potential of light-shielding effect (where excessive catalyst particles scatter or absorb light and limit photon penetration to the catalyst’s active sites),^43^ 50 mg is selected as the optimal dose for further experimental studies. This dose ensures efficient photocatalytic activity without the drawbacks associated with higher catalyst concentrations.

The initial solution pH is a crucial factor in the degradation of organic contaminants because the charge of most organic compounds changes with pH, and it strongly affects the electrical properties, such as the surface charge of many photocatalysts,^44^ see Figure 4B above. Figure 10 illustrates photodegradation of SMX, TET, and TC in the presence of GCN@ 1:2 vs pH. At pH 3–9, the photocatalyst shows 99% degradation efficiency for SMX (Figure 10A). This could indicate that there is no strong pH dependence in its degradation pattern until pH 9. Although the pHpzc of GCN@ 1:2 is 6.1, the development of surface charge above this value is gradual rather than abrupt. Thus, between pH 7 and 9, the catalyst surface is only moderately negative, which allows partially deprotonated SMX species to still adsorb effectively through nonelectrostatic interactions such as hydrogen bonding and π–π stacking.^45^ This explains the minimal change in degradation efficiency up to pH 9 despite being above the pHpzc. In contrast, at pH 11, the degradation efficiency of the photocatalyst is significantly reduced, even though some degradation is still observed. This can be attributed to the deprotonation of SMX to its anionic form at high pH.^46,47^ Simultaneously, the surface of the photocatalyst is also negatively charged because pH 11 > pH_pzc_ = 6.1, resulting in electrostatic repulsion between the negatively charged catalyst surface and the deprotonated SMX molecules. This repulsion reduces the SMX interaction with the catalyst, thereby hindering its degradation. As a result, only 40% degradation of SMX is observed at pH 11.

**Figure 10:**
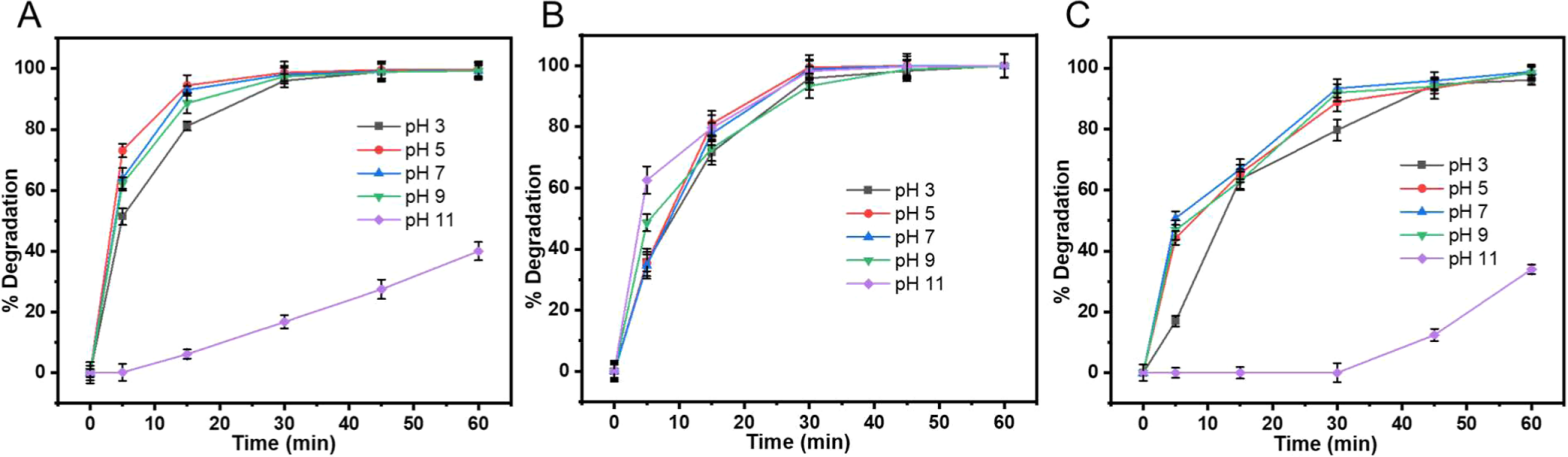
Effect of the pH on (a) SMX, (b) TET, and (c) TC degradation with GCN:Zn@1:2.

On the other hand, TET is an amphoteric compound, and its species exist as TET^+^ (p*K*_a1_ = 3.3), TET^0^ (p*K*_a2_ = 7.7), and TET^–^ (p*K*_a3_ = 9.7).^48^ As a result of the presence of differently charged species at different pH values and a correspondingly different surface charge on the photocatalyst (Figure 4B) versus pH, one could expect a rather strong pH dependence of TET degradation. Interestingly, this is not the case, and TET degradation does not correlate with pH (Figure 10B) throughout the observed pH range. This could suggest that the surface charge-dependent electrostatic forces are not a key parameter for the degradation of TET, similar to another recent study.^44^

For TC (Figure 10C), the photocatalyst shows a 99% degradation efficiency between pH 3 and 9, similar to SMX. Although pH 7–9 is above the pHpzc (6.1), the gradual development of negative surface charge in this range means the catalyst is only moderately negative, allowing partially deprotonated TC species to still adsorb effectively via hydrogen bonding and π–π interactions. This explains the stable degradation up to pH 9 despite a change in surface charge polarity.

However, TC degradation is also significantly affected at pH 11, where it exists in its deprotonated anionic form.^46,47^ The negatively charged photocatalyst surface at pH 11 creates electrostatic repulsion with TC, reducing its adsorption onto the catalyst surface and limiting its degradation efficiency. Consequently, while 99% TC removal is achieved at pH 3–9, only 30% removal occurs at pH 11 after 60 min.

The influence of the initial PPCP concentration on their degradation is shown in Figure 11A. Overall, the degradation efficiency of GCN:Zn@1:2 decreases slightly from 100% to ca. 98% as the initial concentration of the contaminants increases from 2 to 20 ppm in 60 min. This very minor reduction in efficiency can be attributed to the fact that simply more PPCP molecules are present in the solution vs a constant number of active catalytic sites producing ROS;^49^ this slightly reduces the degradation efficiency. As the number of ROS generated is roughly constant even at higher PPCP concentrations, the PPCP/ROS ratio very slightly shifts.

**Figure 11:**
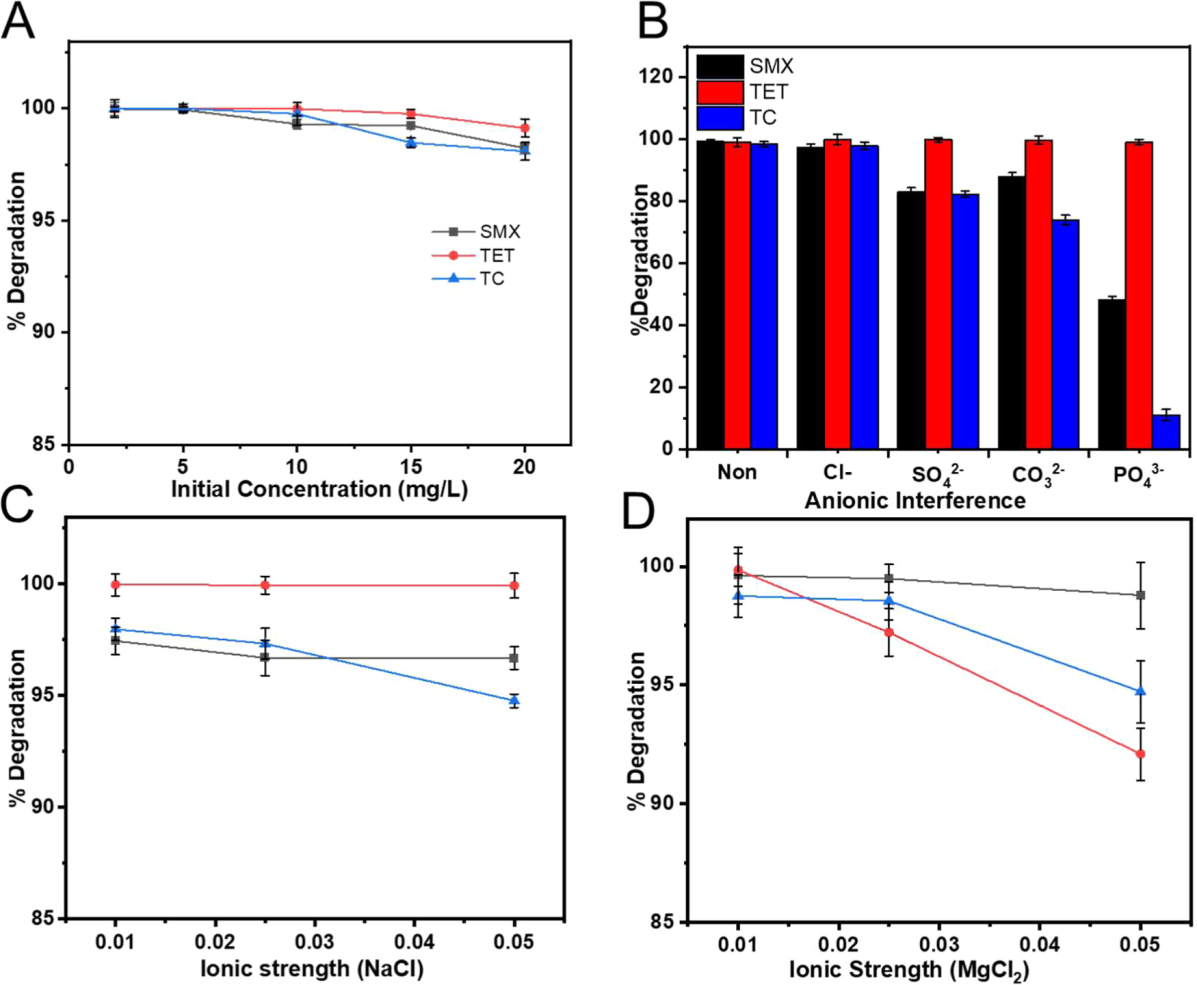
(A) Effect of initial PCPP concentration at a fixed catalyst dose, (B) effect of anions, (C) effect of ionic strength with NaCl, and (D) effect of ionic strength with MgCl_2_ on PPCP degradation with GCN:Zn@1:2.

Moreover, at higher PPCP concentrations, the increased optical density of the solution can limit the penetration of photons to the photocatalyst, mostly by absorption.^50^ This may also slightly reduce the generation of ROS and, therefore, contribute to the observed effect. Overall, however, GCN:Zn@1:2 demonstrated an excellent efficiency for PPCP degradation even at elevated PPCP concentrations.

The influence of the anions present in the system is shown in Figure 11B. The results show that the presence of phosphate (PO_4_^3–^), sulfate (SO_4_^2–^), and carbonate (CO_3_^2–^) leads to a reduction in the degradation efficiency of SMX and TC, whereas the degradation of TET remains unaffected. The reduction in degradation efficiency for SMX and TC suggests that these anions interfere with the photocatalytic process by competing for active sites on the surface of the photocatalysts.

This behavior can be explained by the adsorption properties of anions, which are primarily influenced by their ionic radii.^51^ In this study, the ionic radii of anions are as follows: PO_4_^3–^ (∼0.31) nm, SO_4_^2–^ (∼0.24 nm), and CO_3_^2–^ (∼0.18 nm) are all smaller than those of the target pollutants; TET, SMX, and TC have radii of ∼0.49, ∼0.55, and ∼0.52 nm. Due to their smaller size and higher charge, PO_4_^3–^, SO_4_^2–^, and CO_3_^2–^ likely occupy active sites on the photocatalyst surface and reduce the availability of space for SMX and TC to adsorb onto the photocatalyst surface and to interact with the generated ROS. In contrast, the degradation of TET remains unaffected, likely because its degradation mechanism does not rely heavily on surface adsorption or is less influenced by competition with these anions.

Besides surface blocking, ionic strength of the solution is another important factor governing the degradation of pollutants.^52^ For example, increasing the ionic strength with a monovalent cation and a monovalent anion (NaCl, Figure 11C) shows no significant effect on the degradation. In contrast, the addition of a divalent cation and monovalent anion (MgCl_2_, Figure 11D) leads to a slight reduction of the PPCP degradation (approximately 98%, 92%, and 95% degradation of SMX, TET, and TC, respectively). This may be attributed to (1) the fact that the higher ionic strength of Mg^2+^ vs Na^+^ could lead to a more significant compression of the electrical double layer around the photocatalyst surface and (2) to the fact that Mg^2+^ has a higher charge density than Na^+^ and can therefore interact more strongly with the photocatalyst surface.^53^ Clearly, Mg^2+^ will have a stronger hydration shell, and some of these effects may therefore be counterbalanced by a slightly higher energy required to release water molecules from around the Mg^2+^ than the Na^+^ ion. That is, Mg^2+^ could be slightly slower to attach to the surface of the catalyst because of the stronger hydration shell, but once attached, it could lead to more effective blocking of active sites on the catalyst surface. The latter effect could lead to some blocking of active sites on the catalyst, reducing its efficiency.

Furthermore, the relatively weak reduction in the TET concentration in the presence of Mg^2+^ can be associated with the fact that TET contains multiple functional groups, such as hydroxyl (–OH) and keto (C=O) groups, that can act as ligands for Mg^2+^. This could possibly lead to chelation complexes with Mg^2+^ ions (TET-Mg^2+^),^54,55^ which may in turn lead to TET being less available for the photocatalytic degradation.

### 3.4 Effect of Scavengers on PPCP Degradation

To identify the role of different ROS types in the PPCP photodegradation a scavenger test was performed using isopropyl alcohol (IPA) as a hydroxyl radical scavenger (^•^OH), benzoquinone (BZQ) as a superoxide scavenger (O_2_^–•^), and sodium oxalate (NaOx) as a hole scavenger (h^+^).^56^ Figure 12A shows that the addition of IPA does not change the degradation of the PPCPs. This indicates that hydroxyl radicals are not the primary reactive species responsible for PPCP degradation.

**Figure 12:**
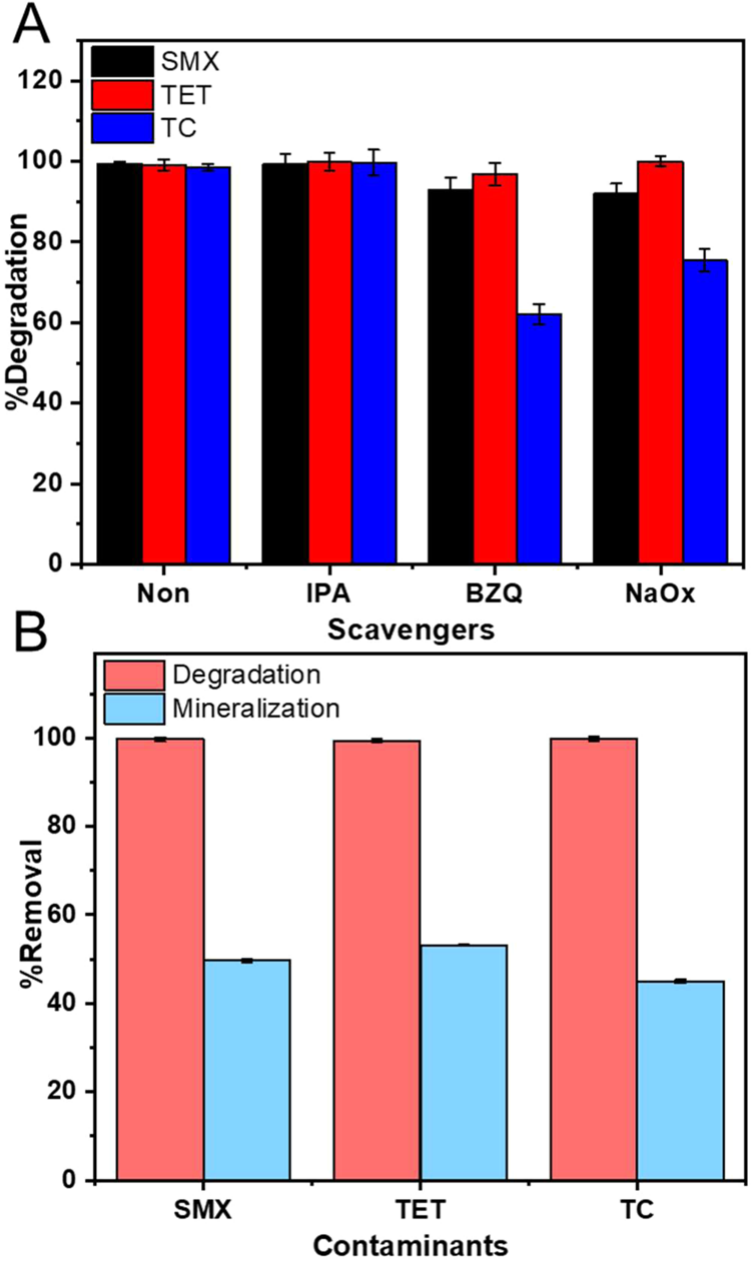
(A) Effect of radical scavengers on PPCP removal and (B) mineralization and degradation of PPCPs in aqueous solution in the presence of GCN:Zn@1:2 under visible light irradiation.

In contrast, the addition of BZQ leads to a slight decrease in the degradation of SMX (92%) and TET (96%), and a more notable decrease in the degradation of TC (62%). This suggests that superoxide radicals are involved in the degradation of all three contaminants, with a particularly significant role in the degradation of TC. Finally, holes also appear to play an important role in the degradation of SMX and TC. This is evidenced by the decrease in the degradation efficiency that is observed when NaOx is added to the reaction mixture.

To further support the conclusion that O_2_^–•^ and h^+^ are the main ROS responsible for the degradation of SMX and TC, the results from the pH studies (Figure 10) can be taken into account: these data show a reduced photocatalytic efficiency for both SMX and TC at pH 11. This can be attributed to the fact that at high pH, superoxide radicals (O_2_^–•^) will transform to the superoxide anions (O_2_^2–^).^57^ The superoxide anion is a much weaker oxidant than the superoxide radical and will therefore show a reduced degradation efficiency vs the more reactive superoxide radical. Furthermore, at high pH, the efficiency of h^+^ is reduced due to either high OH^–^ concentration competing with photogenerated holes, or electrostatic repulsion between the negatively charged catalyst surface and the contaminants, which lowers the overall degradation efficiency.

### 3.5 Mineralization of PPCPs

The efficiency of GCN@1:2 for the mineralization of SMX, TET, and TC was evaluated via total organic carbon (TOC) analysis of the treated water, as shown in Figure 12B. Under visible light irradiation, GCN@1:2 mineralizes ca. 50% (SMX), 53% (TET), and 45% (TC), whereas over 97% degradation efficiency is obtained for all contaminants; see Figure 10. The mineralization efficiency for all three contaminants is therefore notably lower than the respective degradation efficiency.

In order to further analyze the intermediates of the degradation reaction, liquid chromatography–mass spectrometry (LC–MS) analysis was done. The intermediate products of SMX degradation mainly involve four intermediates (for MS data, see Figures S3 and S4A–D, Supporting Information). Initially, a well-defined peak at retention time (rt) 11.660 min is ascribed to SMX. Figure 13 shows that two of the intermediates with mass-to-charge (*m*/*z*) ratios [178.04 (rt 4.89 min) and 162.01 (rt 2.88 min)] appear before the rt of SMX, which suggests that the compounds corresponding to these two signals are more polar than the starting compound SMX. However, the other two intermediates [283.10 (rt 14.99 min) and 226.04 (rt 12.69 min)] appear after the rt of SMX, suggesting the formation of two less polar intermediates. The same fragments with *m*/*z* 283.10, 226.04, 178.04, and 162.01, have been observed previously.^58–60^ These intermediates from the degradation of SMX were further degraded to their inorganic components, such as CO_2_, H_2_O, and SO_4_^2–^.

**Figure 13:**
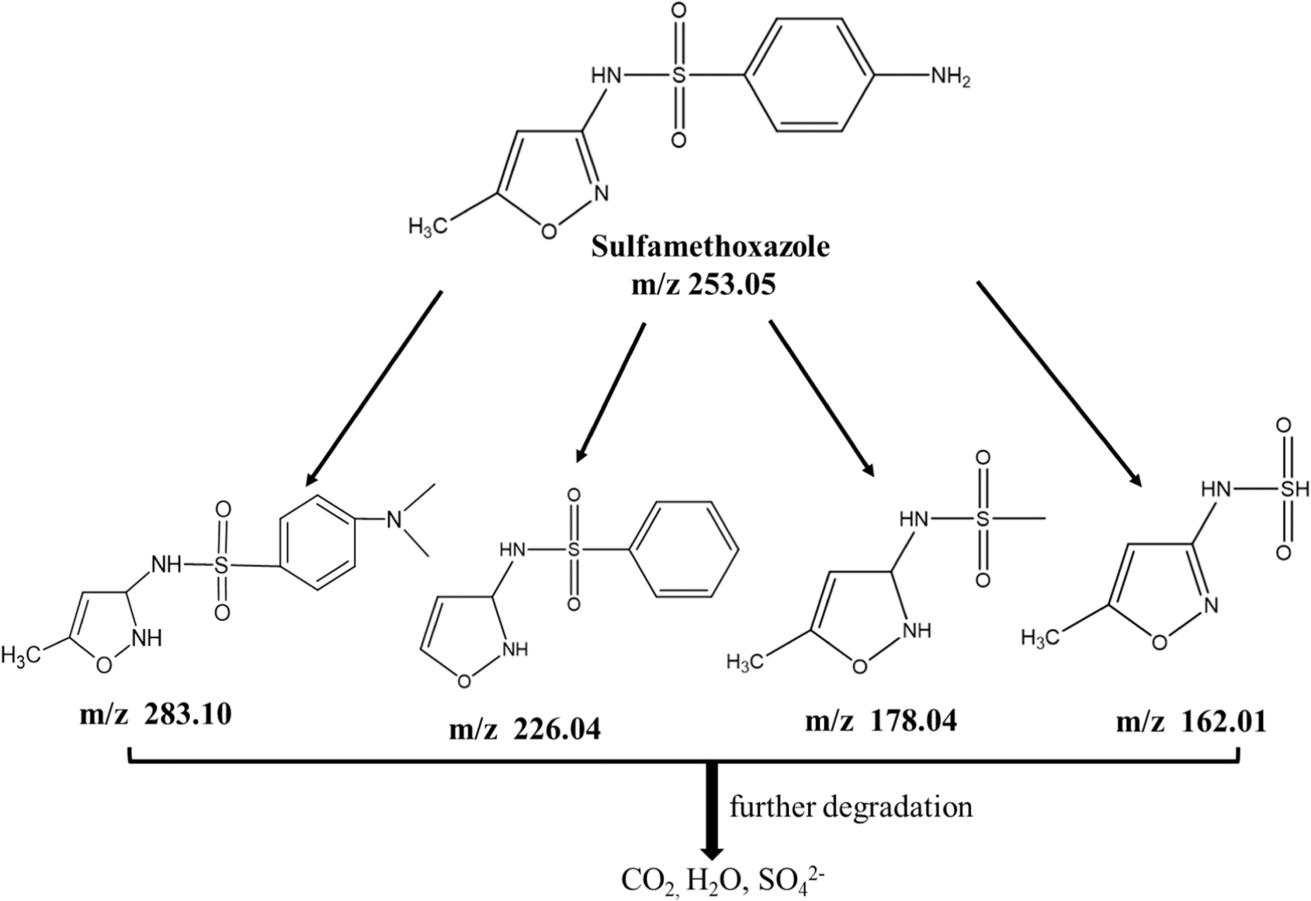
Proposed structures of degradation intermediates of SMX identified by mass spectrometry.

For TET degradation, the LC–MS data (Figure S3A,E) show the formation of four intermediate products. The primary characteristic peak, with a rt of 12.71 min and *m*/*z* of 445.16 corresponds to the deprotonated TET molecule ion. The degradation of TET could possibly occur by the attack of reactive species, leading to structural modifications through pathways including dehydroxylation, functional group cleavage, and ring opening.^61^ Figure 14 shows the four main intermediates with *m*/*z* of 285.14 (rt = 2.47 min), 170.09 (rt = 2.48 min), and 130.12 (rt = 5.65 min) after photodegradation. According to Figures S5 and S6A–D, the retention time of the four intermediates is shorter than that of TET, indicating that more polar intermediates are formed during the photocatalytic process.^62^ The same fragments with *m*/*z* 285.14, 170.09, 154.06, and 130.12 have been previously reported.^63,64^ Finally, these intermediates were ultimately converted to H_2_O, CO_2_, or other molecules through further mineralization

**Figure 14:**
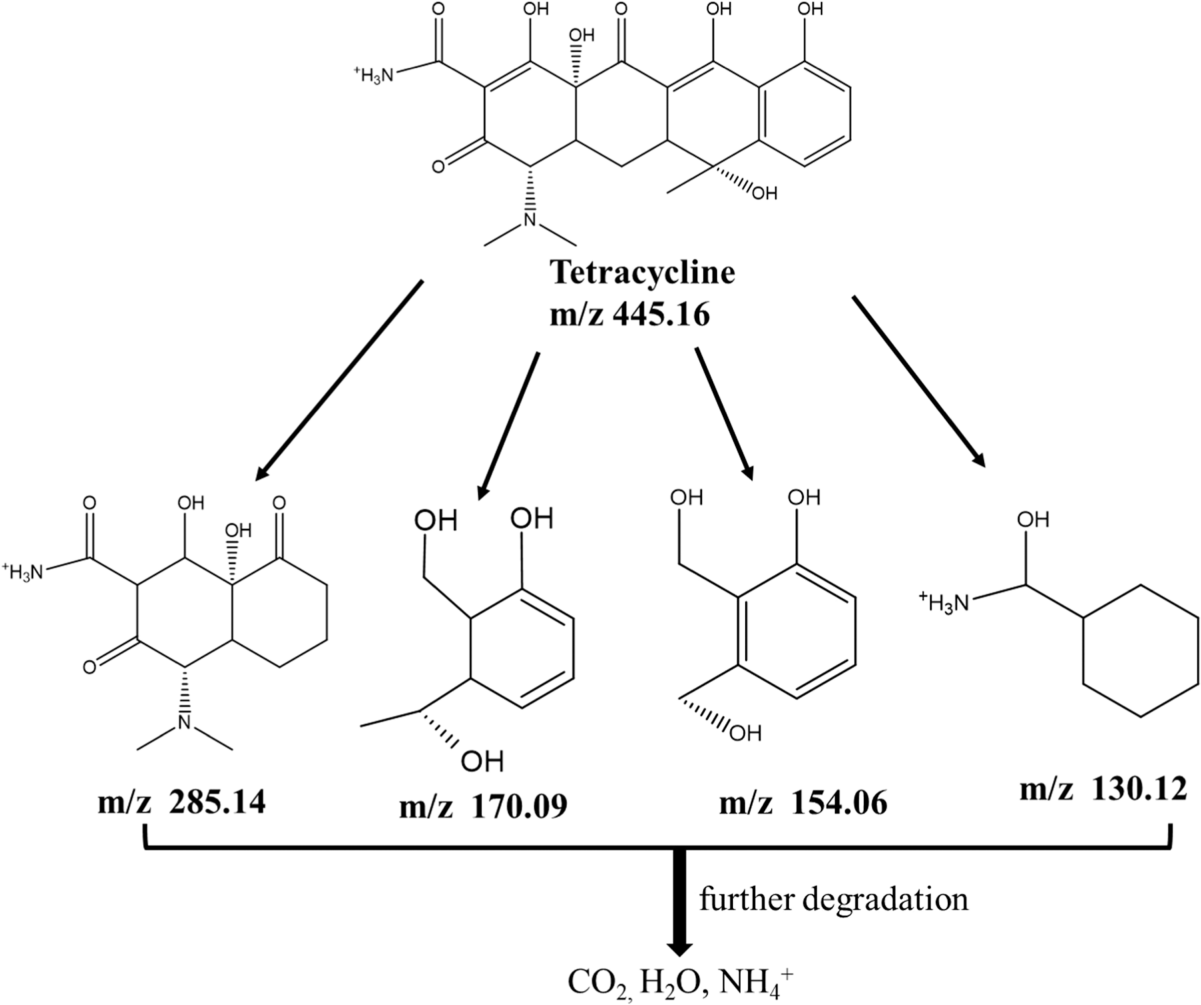
Proposed structures of TET degradation intermediates identified by mass spectrometry.

Finally, the LC–MS analysis of TC shows no intermediates for TC. This most likely indicates that the intermediates in this process cannot be picked up by LC or MS under the experimental setting used in this study.

### 3.6 Toxicity Assessment of Treated Water

Due to incomplete mineralization (Figures 9 and 11B) during the photocatalytic treatment, there is a possibility of producing photodegradation products with higher human and environmental toxicity than the original solutions. To examine whether or not the degradation products of the PPCPs studied here are degraded into toxic or less toxic fragments, the toxicity of the treated solutions was investigated against a Gram-negative (*E. coli*) and a Gram-positive (*S. xylosus*) bacterial strain.

Figure 15 shows that all starting materials (*t* = 0 min; 10 mg/L SMX, TET, and TC) lead to a clear growth inhibition around the paper disk. While no zones of inhibition diameters are observed on *E. coli* plates after the photodegradation of SMX and TET molecules using GCN:Zn@1:2, indicating that the degradation products do not exhibit any higher toxicity toward the tested bacterial strains. However, for TC treatment, a 5 mm zone of inhibition is observed at 15 min, which suggests that ca. 1 mg/L TC is remaining (Table S1, see reference plate). With further treatment beyond 15 min, no zones of inhibition are observed.

**Figure 15:**
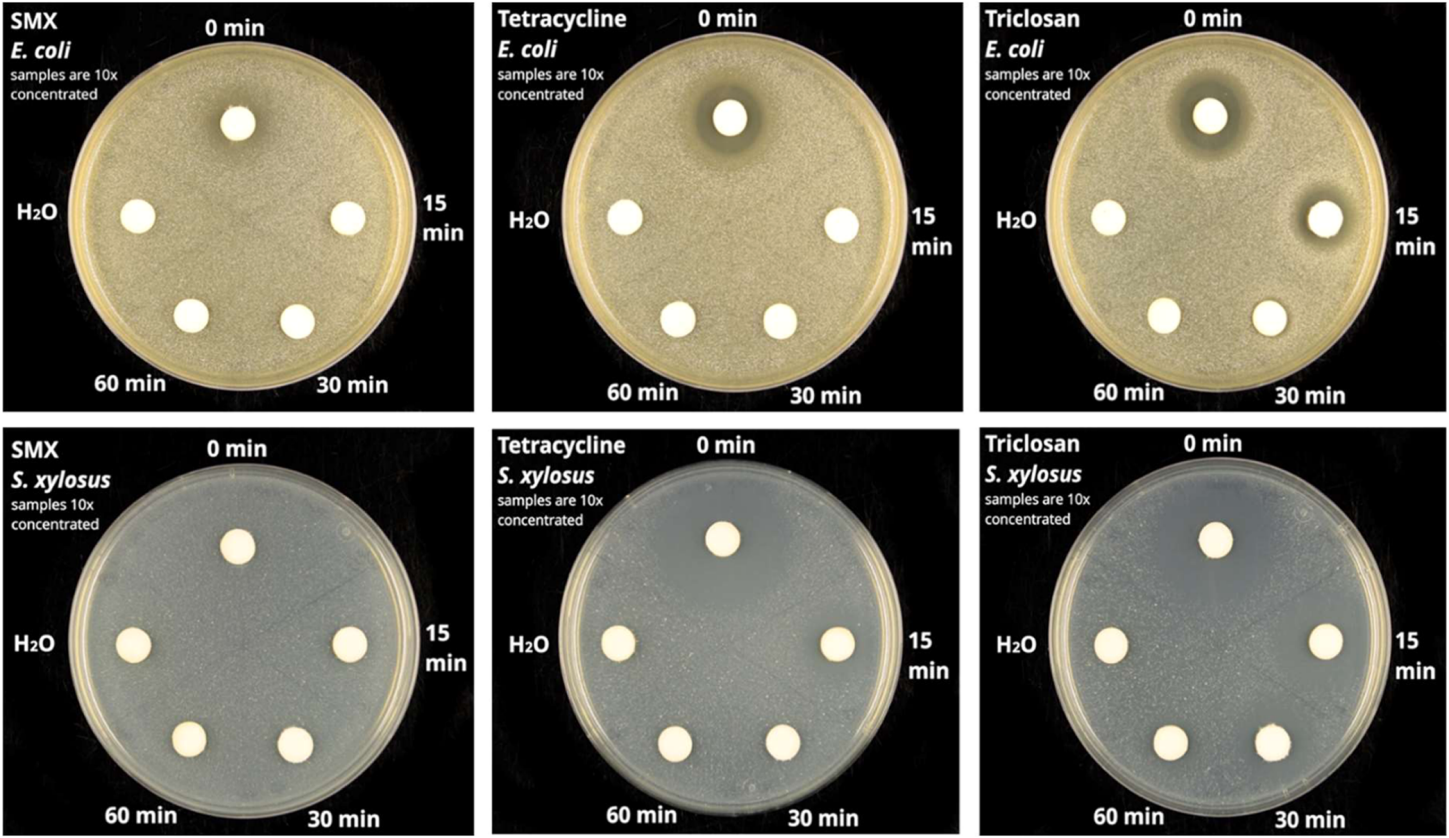
Toxicity assessment for untreated (*t* = 0) and treated water collected at different time intervals for *E. coli* (upper panel) and *Staphylococcus xylosus* (lower panel).

On the other hand, in the case of *S. xylosus*, SMX does not inhibit the growth of the bacteria even at 0 min (i.e., the untreated water). This can be attributed to the fact that SMX is unreactive to *S. xylosus*. For TET, a zone of inhibition was observed at 15 min, and after 15 min of treatment, there was a loss of inhibition zone. While for TC, a zone of inhibition of about 6 mm was observed until 30 min, indicating that ca. 1 mg/L (Table S1, see reference plate) of TC is left in the sample.

### 3.7 Reusability of the Photocatalysts

The reusability of the GCN:Zn@1:2 photocatalyst was investigated by subjecting it to four consecutive cycles of degradation experiments for SMX, TET, and TC under the same conditions as previously described (Section 2.3). As presented in Figure 16A, the photocatalytic degradation of all contaminants (SMX, TET, and TC) remain consistent, reaching over 98% within 60 min after four cycles, with only a minor decrease in activity observed during the fourth cycle. This slight decrease in efficiency is attributed to the possible loss of photocatalyst mass during the recollection and washing process between cycles.

**Figure 16:**
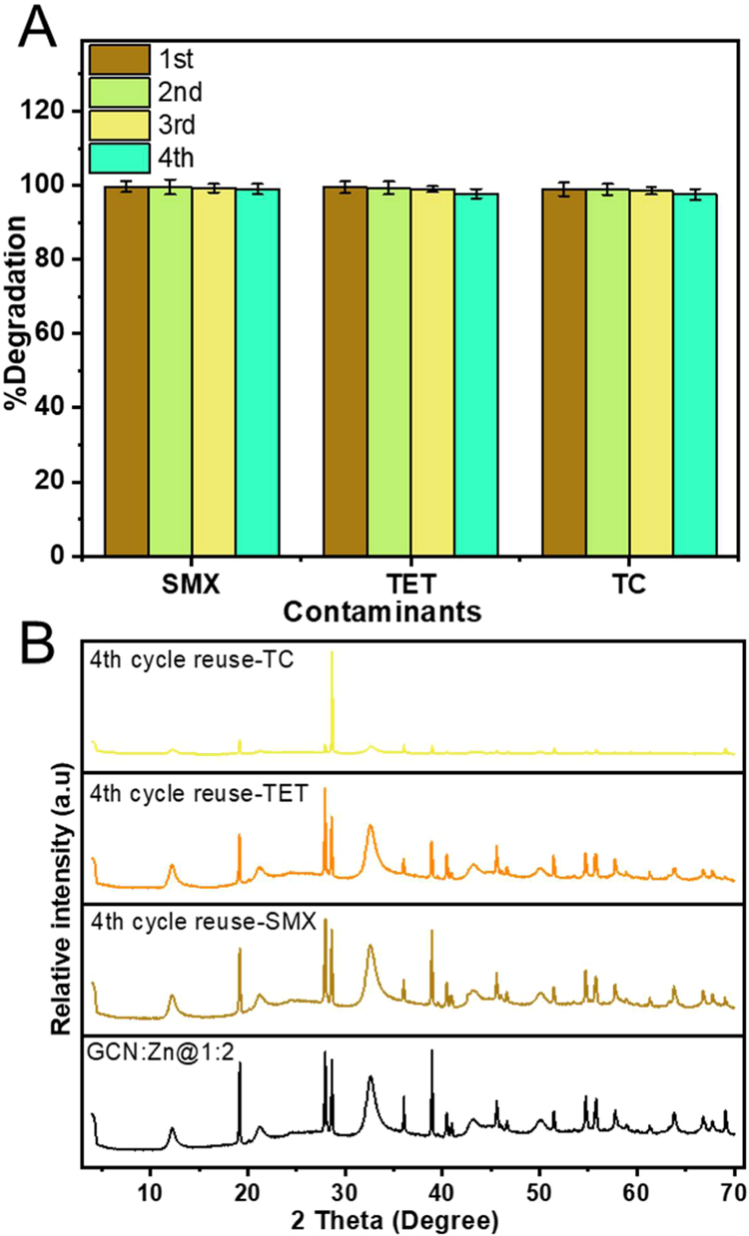
(A) Percentage of PPCP (SMX, TET and TC) degradation upon GCN:Zn@1:2 recycling and (B) XRD patterns.

Additionally, factors such as partial deactivation of active sites and possible blockage of reactive sites by accumulated intermediates over repeated uses could contribute to this decrement.^17,65^ However, in spite of the slight decrease in activity, the data clearly show that GCN:Zn@1:2 can be reused at least four times without losing its photocatalytic activity.

The stability of the photocatalyst was further confirmed by XRD. Figure 16B shows the XRD patterns of the GCN:Zn@1:2 photocatalyst before and after the photodegradation experiments. The XRD reflections of the materials exposed to SMX and TET are essentially identical to those of the starting material and demonstrate a good stability of the material. In the case of TC exposure, the XRD pattern shows a shift in the intensity with the (002) reflection at 28.6° becoming much more intense than in the starting material. Likely this intense 002 reflection indicates that the material may have rearranged to produce a better crystalline order along the crystallographic *c-*axis of GCN in GCN:Zn@1:2. Overall, XRD shows that that the GCN:Zn@1:2 photocatalyst is stable and can be used as a promising material for the photodegradation of PPCP.

## 4 Comparison of GCN:Zn@1:2 with Other Graphitic Carbon Nitride Composites

Over the recent past, a number of graphitic carbon nitride photocatalysts have been developed, and Table 3 shows some of the key parameters of those materials in comparison to GCN:Zn@1:2. Overall, Table 3 shows that the new GCN:Zn@1:2 photocatalyst shows great promise for the degradation of PPCPs. Its performance is comparable and, in some cases, better than previously reported materials. The advantages of GCN:Zn@1:2 include:

(1) a simple solvent-free synthesis,
(2) a low-energy source of illumination,
(3) high degradation efficiency for different contaminants,
(4) high surface area providing abundant reaction sites and finally
(5) a high recyclability and stability over several reuse cycles

**Table 3:** Comparison of GCN:Zn@1:2 with Other Graphitic Carbon Nitride-Based Photocatalysts

photocatalyst	contaminant	photodegradation time (min)	photocatalyst dose (g)	contaminant concentration (mg/L)	surface area (m^2^/g)	photocatalytic degradation efficiency (%)	mineralization (%)	reference
IK-C_3_N_4_	SMX	45	2	10	93.46	99		^66^
FeOy/S-g-C_3_N_4_	SMX	90	0.5	10	7.4	71.5		^59^
pNS-g-C_3_N_4_	SMX	30	0.02	0.5	0.0105	93.7		^32^
0.03 Bi-CNNS	TET	30	0.01	20	90.99	94.1	82.7	^67^
5% CN@BO	TET	50	0.05	10		80.2	59.2	^68^
g-C_3_N_4_-MnFe_2_O_4_ (1:1)	TC	60	0.2	9	98.932	95	44.6	^69^
LNRs/GCN-NRs	TC	90	0.05	10		99.9		^70^
S-Ag/TiO_2_@g-C_3_N_4_	TC	60	0.2	10		92.3		^71^
GCN:Zn@1:2	SMX	60	0.05	10	150.17	99.3	50	this study
GCN:Zn@1:2	TET	60	0.05	10	150.17	99.5	53	this study
GCN:Zn@1:2	TC	60	0.05	10	150.17	98.9	45	this study

## Conclusions

This study demonstrates the successful development of a porous GCN photocatalyst doped with Zn via a solvent-free manual solid-state mixing synthesis, providing an environmentally friendly and scalable method for photocatalyst fabrication. The method aligns with green chemistry principles, offering a scalable, cost-effective, and environmentally friendly route for photocatalyst fabrication. The incorporation of zinc significantly enhances the structural and electronic properties of GCN, including increased surface area, improved porosity, and possibly a faster charge transfer to the organic substrates, resulting in remarkable photocatalytic performance for PPCP degradation under visible light. The optimized GCN:Zn@1:2 composite achieves nearly complete degradation (≥98%) of SMX, TET, and TC within 60 min. Superoxide radicals (O_2_^–•^) and photogenerated holes (h^+^) are the dominant contributors to the degradation process. However, total organic carbon (TOC) analysis indicates only moderate mineralization efficiencies (45–53%). This is attributed to the formation of stable intermediates and low molecular weight fragments. The photocatalyst exhibits excellent reusability, maintaining over 98% degradation efficiency across four cycles with no loss in structural integrity. Toxicity assessments further confirm the safety of the treated water and highlight the potential of these new hybrid photocatalysts for low-cost photocatalytic water treatment under visible light conditions.

## Supplementary Material



## Notes

Reference diffraction pattern of zinc cyanamide (Figure S1); BET plot for the prepared photocatalysts (Figure S2); Ms data of SMX (Figure S3); degradation intermediates of SMX (Figure S4); Ms data of TET (Figure S5); degradation intermediates of TET (Figure S6); and comparison of growth inhibition of water samples after different incubation times with GCN:Zn@1:2 (Table S1) (PDF)

## ◆

Deceased on March 26, 2025.
