# Supplementary material for: Zn-Doped Porous Graphitic Carbon Nitride: A High-Performance Catalyst for the Photodegradation of Pharmaceuticals and Personal Care Products
Source: ACS Omega. 2025 Sep 8;10(36):41395–412. doi: 10.1021/acsomega.5c04537 (PMC12444592; doi:10.1021/acsomega.5c04537)
Supplement: Supplementary file 1 [file ao5c04537_si_001.pdf]

Zn-doped Porous Graphitic Carbon Nitride: A High-Performance Catalyst for the Photodegradation of Pharmaceuticals and Personal Care Products

Chidinma G. Olorunnisola[1,2,*], Damilare Olorunnisola[1,2,3], Christian Neumann[4], Wouter Koopman[5], Christina Günter[6], Harald Seitz[7], Harshadrai M. Rawel[8], Emmanuel I. Unuabonah[2,3*], Andreas Taubert[1,*]

[1] *Institute of Chemistry, University of Potsdam, D-14476 Potsdam, Germany*

[2] *African Centre of Excellence for Water and Environment Research (ACEWATER), Redeemer's University, PMB 230, Ede, Osun State, Nigeria*

[3] *Department of Chemical Sciences, Redeemer's University, PMB 230, Ede, Osun State, Nigeria*

[4] *Fraunhofer-Institut für Angewandte Polymerforschung IAP, D-14476 Potsdam, Germany*

[5] *Institute of Physics and Astronomy, University of Potsdam, D-14476 Potsdam, Germany*

[6] *Institute of Geosciences, University of Potsdam, D-14476 Potsdam, Germany*

[7] *Fraunhofer Institute for Cell Therapy and Immunology, Branch Bioanalytics and Bioprocesses (IZI-BB), D-14476 Potsdam, Germany*

[8] *Institute of Nutritional Science, University of Potsdam, D-14558 Nuthetal, Potsdam, Germany*

Corresponding Authors

Chidinma G. Olorunnisola; ugwujac@run.edu.ng;

Andreas Taubert ataubert@uni-potsdam.de

*E.I Unuabonah Deceased on March 26, 2025

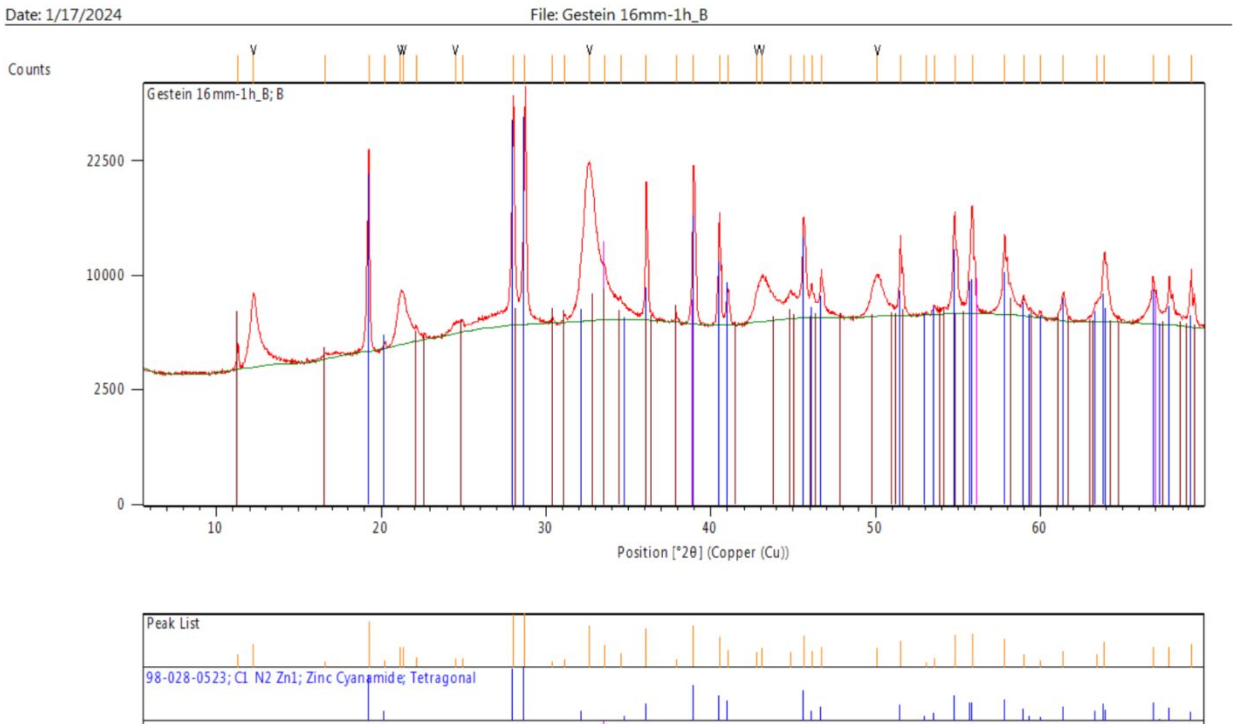

Figure S1: Reference diffraction pattern of zinc cyanamide (ICSD 98-028-0523)

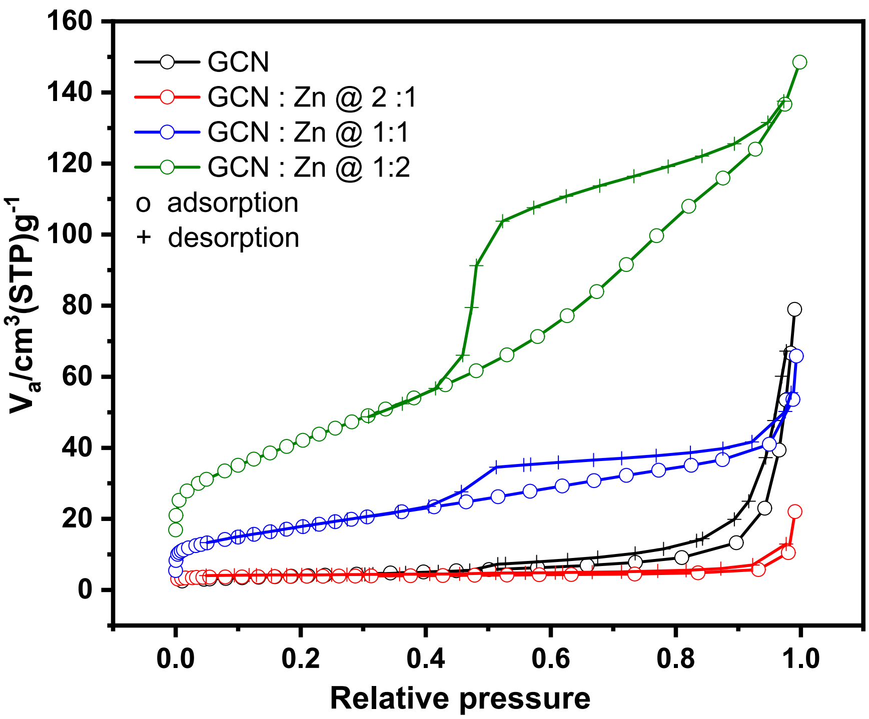

Figure S2: BET plot for the prepared photocatalysts

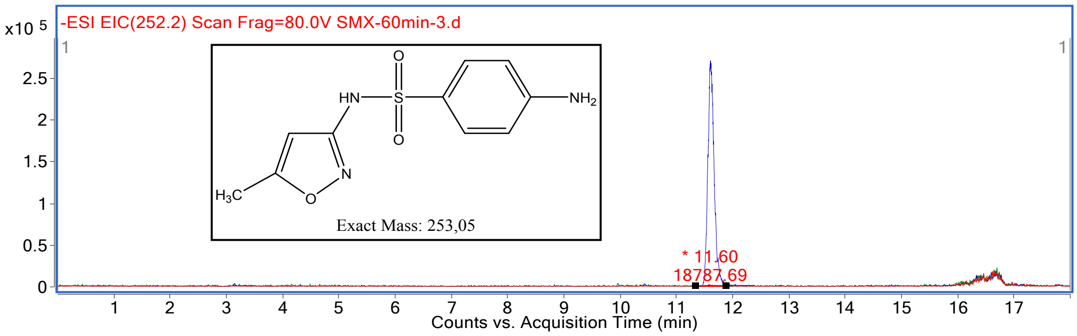

Figure S3: Ms-data of SMX (m/z = 253.05)

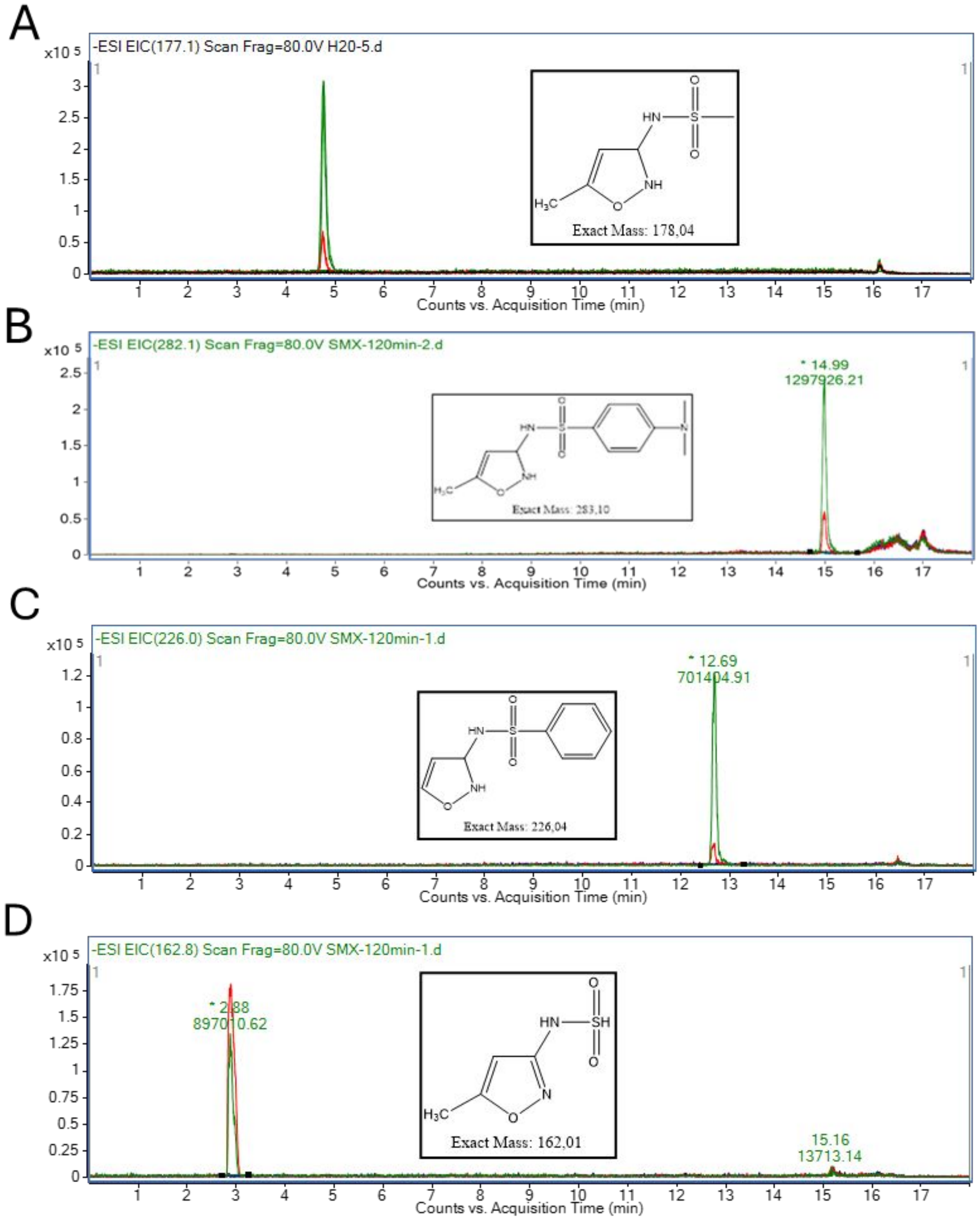

Figure S4: Degradation intermediates of SMX (A) m/z = 253.05, (B) m/z = 283.10, (C) m/z = 226.04 and (D) m/z = 162.01

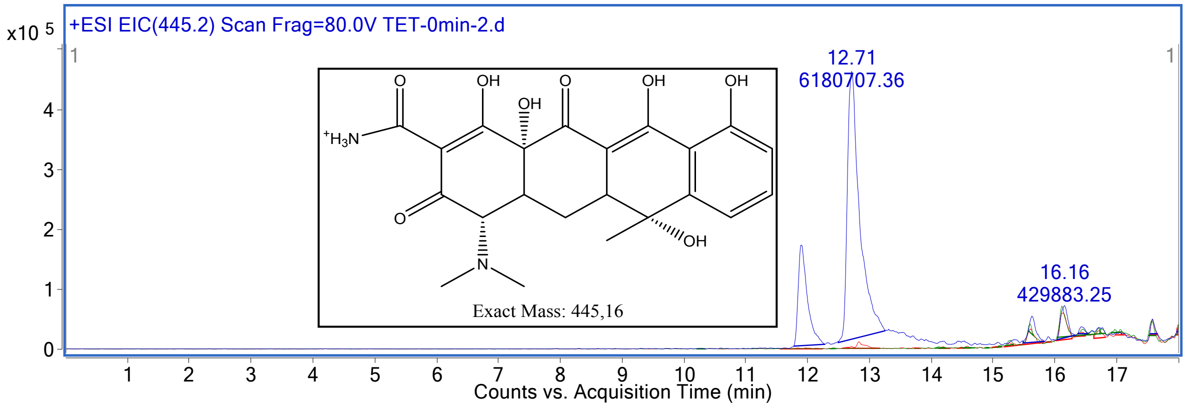

Figure S5: Ms data of TET (m/z = 445.16)

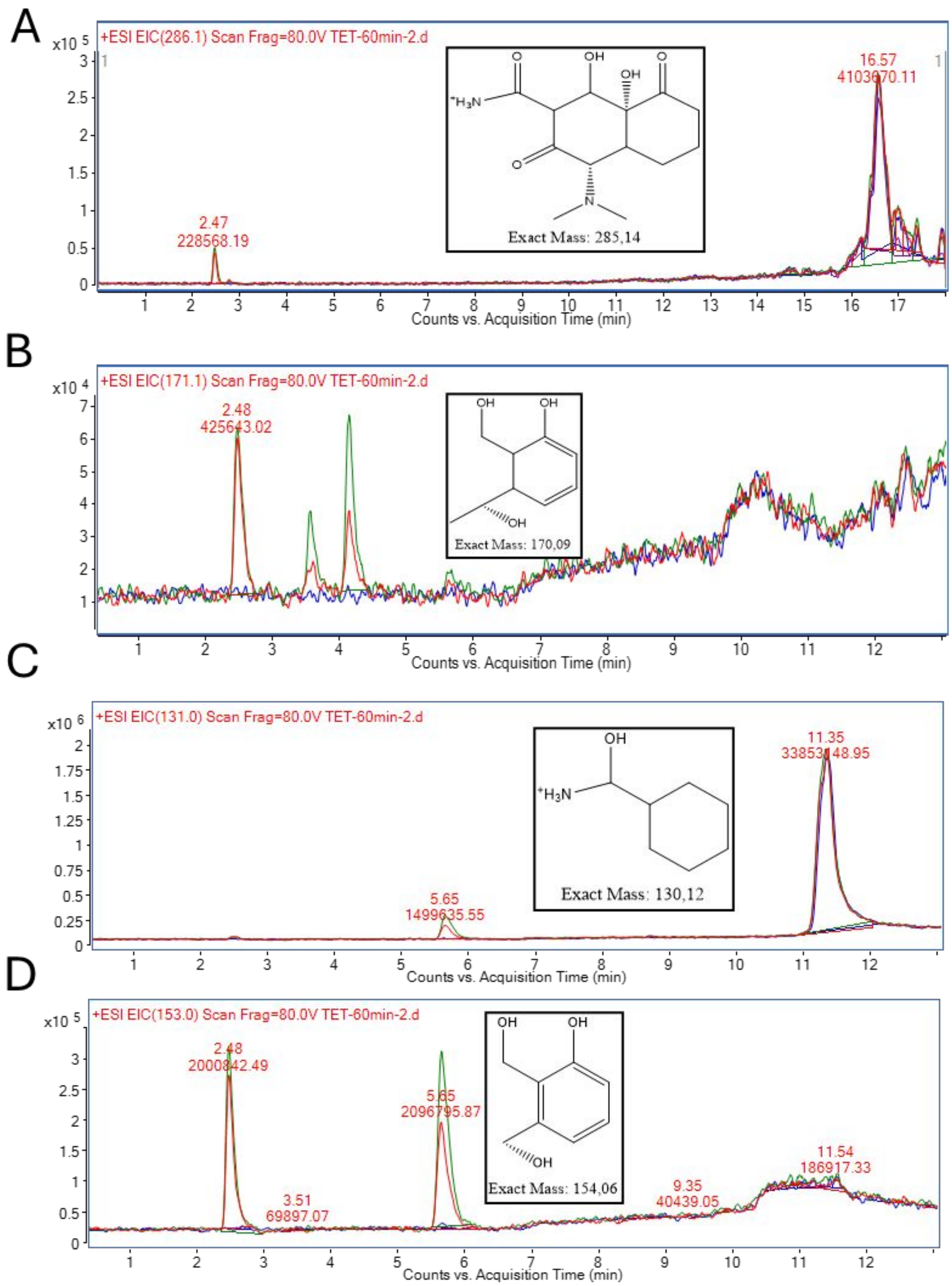

Figure S6: Degradation intermediates of TET (A) m/z = 285.14, (B) m/z = 170.09, (C) m/z = 130.12 and (D) m/z = 154.06

Table S1: Comaparison of growth inhibition of water samples after different incubation times with GCN:Zn @ 1:2

		Plate	Sample	Radius of inhibition area [mm] (radius additional to paper disc)
Tetracyclin	*E.coli*	Reference plate*	10 mg/L	2
			7.5 mg/L	1.5
			5 mg/L	-
			3 mg/L	-
			2 mg/L	-
			1 mg/L	-
			H_2O	-
		Plate with 10x concentrated process samples	0 min	4 – 7.5
			15 min	-
			30 min	-
			60 min	-
			H_2O	-
	S. xylosus	Reference plate*	10 mg/L	6.5
			7.5 mg/L	5.5
			5 mg/L	4
			3 mg/L	3
			2 mg/L	1.5
			1 mg/L	0.5
			H_2O	-
		Plate with 10x concentrated process samples	0 min	13.5
			15 min	4.5
			30 min	1
			60 min	nd
			H_2O	-

**Triclosan**	*E.coli*	Reference plate*	10 mg/L	3 – 5
**Triclosan**	*E.coli*	Reference plate*	7.5 mg/L	1.5
**Triclosan**	*E.coli*	Reference plate*	5 mg/L	-
**Triclosan**	*E.coli*	Reference plate*	3 mg/L	-
**Triclosan**	*E.coli*	Reference plate*	2 mg/L	-
**Triclosan**	*E.coli*	Reference plate*	1 mg/L	-
**Triclosan**	*E.coli*	Reference plate*	H_2O	-
**Triclosan**	*E.coli*	Plate with 10x concentrated process samples	0 min	7.5
**Triclosan**	*E.coli*	Plate with 10x concentrated process samples	15 min	5
**Triclosan**	*E.coli*	Plate with 10x concentrated process samples	30 min	1
**Triclosan**	*E.coli*	Plate with 10x concentrated process samples	60 min	-
**Triclosan**	*E.coli*	Plate with 10x concentrated process samples	H_2O	-
**Triclosan**	*S. xylosus*	Reference plate*	10 mg/L	6
**Triclosan**	*S. xylosus*	Reference plate*	7.5 mg/L	4
**Triclosan**	*S. xylosus*	Reference plate*	5 mg/L	2
**Triclosan**	*S. xylosus*	Reference plate*	3 mg/L	1.5
**Triclosan**	*S. xylosus*	Reference plate*	2 mg/L	-
**Triclosan**	*S. xylosus*	Reference plate*	1 mg/L	-
**Triclosan**	*S. xylosus*	Reference plate*	H_2O	-
**Triclosan**	*S. xylosus*	Plate with 10x concentrated process samples	0 min	11
**Triclosan**	*S. xylosus*	Plate with 10x concentrated process samples	15 min	7.5
**Triclosan**	*S. xylosus*	Plate with 10x concentrated process samples	30 min	6
**Triclosan**	*S. xylosus*	Plate with 10x concentrated process samples	60 min	2
**Triclosan**	*S. xylosus*	Plate with 10x concentrated process samples	H_2O	-
**SMX**	*E.coli*	Plate with 10x concentrated process samples	0 min	2.5
**SMX**	*E.coli*	Plate with 10x concentrated process samples	15 min	-
**SMX**	*E.coli*	Plate with 10x concentrated process samples	30 min	-
**SMX**	*E.coli*	Plate with 10x concentrated process samples	60 min	-
**SMX**	*E.coli*	Plate with 10x concentrated process samples	H_2O	-

	S.xylosus	Plate with 10x concentrated process samples	0 min<br>15 min<br>30 min<br>60 min<br>H_2O	-<br>-<br>-<br>-<br>-
